# ht-MASH: a high-throughput, cost-effective, and robust protocol for microscopic 3D imaging of human angio- and cytoarchitecture in large human brain samples

**DOI:** 10.1007/s12565-025-00859-w

**Published:** 2025-07-08

**Authors:** Sven Hildebrand, Johannes Franz, Shubharthi Sengupta, Anna Schueth, Andreas Herrler, Alard Roebroeck

**Affiliations:** 1 Department of Cognitive Neuroscience, Faculty of Psychology and Neuroscience, Maastricht University (UM), Maastricht, The Netherlands; 2 Department of Anatomy and Embryology, Faculty of Health, Medicine, and Life Sciences, Maastricht University (UM), Maastricht, The Netherlands; 3 Present Address: Microscopy Core Lab, Faculty of Health, Medicine, and Life Sciences, Maastricht University, Maastricht, The Netherlands; 4 Present Address: ASML, Veldhoven, The Netherlands; 5 Present Address: Faculty of Health, Medicine, and Life Sciences, Genetics and Cell Biology, NUTRIM-Institute of Nutrition and Translational Research in Metabolism, GROW-Research Institute for Oncology and Reproduction, Maastricht University, Maastricht, The Netherlands

**Keywords:** Optical tissue clearing, 3D histology, Human neocortex, Cytoarchitecture, Angioarchitecture

## Abstract

The performance of many optical tissue clearing protocols has considerably improved in the last few years, so that now even notoriously difficult specimen such as highly myelinated human brain tissue can be rendered highly transparent. However, optical tissue clearing is still routinely performed on relatively small samples, especially in the case of the human brain. Recent advances in histological tissue processing now allow scaling up the clearing process considerably towards much larger samples. Yet so far, these methods can have considerable drawbacks in their feasibility to be implemented routinely, especially in smaller laboratories. Here, we present an updated version of our MASH protocol, which allows optical tissue clearing of very large human brain tissue samples and labelling of angio- and cytoarchitecture therein. This pipeline is cost-efficient and easy to implement, so that even smaller labs can apply it at scale. At the same time, the use of rapid prototyping using 3D printing to create custom clearing equipment is versatile enough to be adjusted to other optical tissue clearing methods than the one used in this study (e.g., aqueous methods such as CUBIC or other solvent-based methods of the DISCO family), sample sizes or tissue types. Our pipeline has, therefore, the potential to advance optical tissue clearing and labelling of large human tissue samples towards a more robust and routine implementation in the blooming field of 3D histology.

**Graphical abstract:**

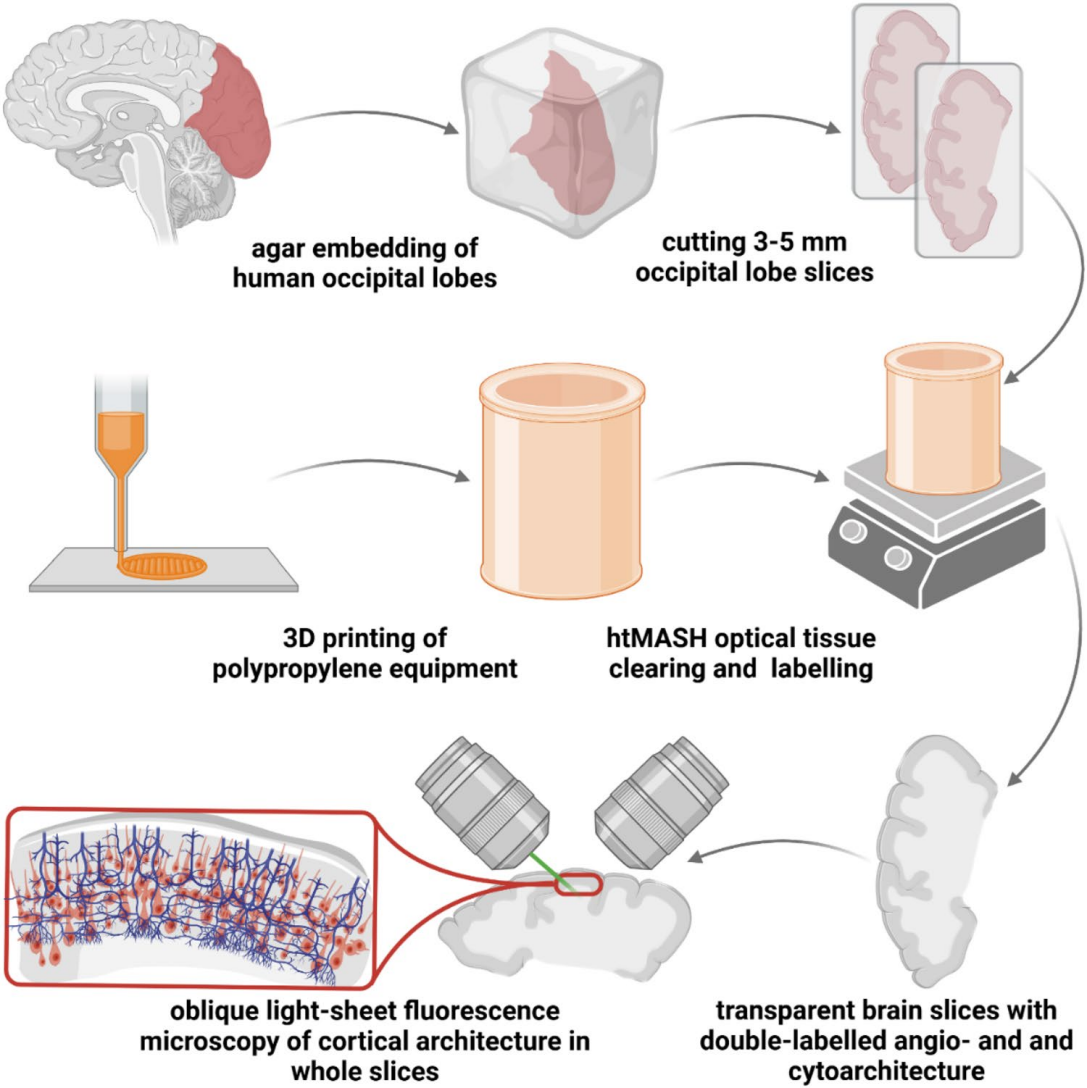

**Supplementary Information:**

The online version contains supplementary material available at 10.1007/s12565-025-00859-w.

## Introduction

Despite the recent advances in histological and imaging methods, the investigation of micro-to-mesoscopic anatomical features of the human brain at mesoscopic scale (many millimetres to centimetres) is still a challenge. Imaging techniques need to have both the resolution to properly visualize these features at the microscopic level but must also cover large extents of tissue to view these features in their spatial context. Until relatively recently, the method of choice to investigate mesoscopic features such as cortical cytoarchitecture was the 3D reconstruction of thousands of 2D brain sections (Amunts et al. 2013, 2020). While this has yielded exceptional results, new 3D histological methods are providing a promising, alternative for this type of investigation, because they have the potential to yield faster and less labour-intense processing pipelines.

Optical tissue clearing (OTC) is rapidly becoming a standard technique for structural investigations of brain (and other) tissue. Until recently, human brain samples where still relatively small in their lateral extent as emphasis was primarily on improving the clearing and labelling towards thicker pieces of tissue (Hildebrand et al. 2019, 2020; Lai et al. 2018; Liebmann et al. 2016; Liu et al. 2016, 2017; Morawski et al. 2018). The lateral size, however, was so far limited mainly by the microscope systems. Conventional light-sheet fluorescence microscopy (LSFM) systems introduce excitation light laterally into the sample. This limits the lateral size of the sample to a few centimetres at most, depending on the excitation wavelength, due to light scattering. New systems that address this particular shortcoming have been introduced recently (Glaser et al. 2017, 2019; Schueth et al. 2023). These systems mostly rely on an objective geometry often referred to as oblique LSFM. Oblique LSFM describes systems, in which both objectives are located either above or below the sample and will often be angled with respect to the tissue surface. This imaging geometry is necessary for very large samples, because the light penetration is ultimately the most limiting factor in data acquisition, even in highly transparent samples. With these new imaging systems, efforts were made by some groups to scale up the histological pipeline towards very large slices of human brain tissue and even entire organs (Ku et al. 2020; Zhao et al. 2020; Park et al. 2024). Even with the new imaging platforms however, the microscopic investigation of entire cleared brains or even slices of more than a centimetre thickness is currently unrealistic and the processing of an entire cleared brain can take over 2 months (Zhao et al. 2020). The elasticising of human brain slices to allow for deep and fast label penetration is a promising approach but this method requires sophisticated equipment and special chemicals, which are not readily available in current histology laboratories. The reliance on elaborate machinery to optically clear the tissue and introduce the labels (Ku et al. 2020; Park et al. 2024) currently makes it difficult for smaller scale laboratories to adapt these methods.

A fast, cost-efficient, easy to implement, and scalable (both in terms of tissue size and throughput) histological pipeline for the 3D investigation of very large brain slices is therefore highly desirable as an additional tool, even if it would be confined to a few common labelling targets. This would also allow smaller laboratories to profit from the full potential of OTC for neuroanatomical studies. Here we present an updated protocol of our recently published MASH approach (Hildebrand et al. 2019), which is based upon an adjusted iDISCO + (Renier et al. 2016) version optimised for human brain tissue, paired with several economic stainings to label cytoarchitecture (Hildebrand et al. 2019; Schueth et al. 2023) and angioarchitecture (Hildebrand et al. 2024). This new high-throughput version has been created with the intention to clear and label entire coronal occipital lobe slices of 3–5-mm thickness. We use custom-made 3D printed clearing containers, compatible with the corrosive organic solvents used in the clearing technique, to accommodate multiple coronal slices. This pipeline can clear and stain multiple (up to 10) large portions of human occipital lobes within 10–18 days in a single iteration. The small molecule labels penetrate deeply into the tissue using only passive diffusion, even in very large samples. We demonstrate the high transparency and staining quality of these samples, by acquiring mesoscopic overview scans with oblique LSFM on two different systems.

## Protocol

### Materials

#### Key resource table

**Table:** 

Reagent or resource	Source	Identifier
Chemicals		
Agar	Merck KGaA (Sigma–Aldrich)	05040-1KG
Citric acid monohydrate	Merck KGaA (Supelco)	1.00244.0500
Dichloromethane	Carl Roth GmbH + Co. KG	8424.2
Disodium phosphate dihydrate	Merck KGaA (Supelco)	1.06580.5000
Ethyl cinnamate	Merck KGaA (Sigma–Aldrich)	W243000-1KG-K
Hydrogen peroxide (30%)	Carl Roth GmbH + Co. KG	P26.4
Methanol	Merck KGaA (Supelco)	1.06009.2511
Potassium chloride	Merck KGaA (Supelco)	1.04936.1000
Potassium disulfite	Carl Roth GmbH + Co. KG	7995.3
Sodium chloride	Thermo Fisher Scientific (Fisher chemical)	AC207790010
Sodium dodecyl sulfate	Carl Roth GmbH + Co. KG	2326.2
Sodium dihydrogen phosphate	Merck KGaA (Supelco)	1.06346.1000
Thymol (optional)	Merck KGaA (Sigma–Aldrich)	16,254-100G
Triton X-100	Merck KGaA (Millipore)	108,603.1000
Labels for angio- and cytoarchitecture		
* Lycopersicon esculentum* lectin DyLight® 649 conjugated	Vector laboratories, Inc	DL-1178
Methylene blue (optional)	Carl Roth GmbH + Co. KG	A514.2
Neutral red	Carl Roth GmbH + Co. KG	T122.1
Lab equipment		
25 ml glass pipettes (optional)	Brand (via VWR international)	27,079
50 ml reaction tubes (optional)	Sarstedt AG & Co. KG (via VWR international)	SARS62.547.254
Costar® 6 well cell culture plates (optional)	Corning (via VWR international)	3516
Rotary meat cutter “Allesschneider contura 3”	ritterwerk GmbH (via amazon)	B001UK0WUM
ct-dSPIM light-sheet microscope	Applied Scientific Instrumentation, Inc	
Magnet stirrer with heating plate	SUNLAB GERMANY (via neoLab Migge GmbH)	D-8150
Orbital shaker (optional)	SUNLAB GERMANY (via neoLab Migge GmbH)	D-8120
Fisherbrand™ PTFE container 2.2 l (optional)	Thermo Fisher Scientific	10,660,473
QuVi SPIM light-sheet microscope (optional)	LUXENDO GmbH	https://www.brukersupport.com/ProductDetail/9248
Adhesives for sample mounting		
Glue gun super/promatic	Bison international B.V	6,311,397
Silicone sealant sanitary	Bison international B.V	6,306,988
Gorilla super glue	Gorilla glue	7,900,301
Gorilla waterproof Caulk & Seal 100% silicone sealant—clear	Gorilla glue	108,360
BONDIC® starter	BONDIC (VIKO UG)	BONSTART1
Software and algorithms		
Bigstitcher	(Hörl et al. 2019)	https://imagej.net/plugins/bigstitcher/
FIJI/imageJ	(Schindelin et al. 2012)	https://imagej.net/software/fiji/
LuxBundle software	LUXENDO GmbH	https://www.bruker.com/de/products-and-solutions/fluorescence-microscopy/light-sheet-microscopes/luxbundle-software.html
ClearMap toolbox	Charly Rousseau (2024) “ChristophKirst/ClearMap2: v2.1.4”. Zenodo. 10.5281/zenodo.12795869	https://github.com/ClearAnatomics/ClearMap
Napari	Sofroniew, N. (2025) “napari: a multi-dimensional image viewer for Python”. Zenodo. 10.5281/zenodo.15029515	https://github.com/napari/napari/tree/v0.6.0a0
Napari-nD-annotator		https://www.napari-amhub.org/plugins/napari-nD-annotator
Amira (version 2020.2)	Thermo Fisher Scientific	https://www.thermofisher.com/nl/en/home/electron-microscopy/products/software-em-3d-vis/amira-software.html
3D printing equipment		
Comgrow 3D printer polypropylene building platform	Comgrow (via amazon.de)	B07JPFZ2BZ
Ender 3 pro 3D printer (discontinued: replaced by Ender 3 V3 series)	Creality	
Fiberlogy polypropylene filament 1.75 mm	Fiberlab S.A. (via amazon.de)	B086FZHTB2
Polypropylene adhesion glue " Magigoo pro PP”	Thought3D Ltd (via Carl Roth GmbH + Co. KG)	27H5.1

#### Recipes and solutions

##### 10 × phosphate buffered saline (PBS) stock (1 l)

Dissolve 80 g of sodium chloride, 2 g of potassium chloride, 17.8 g disodium phosphate dehydrate, and 2.4 g Sodium dihydrogen phosphate in 800 ml distilled water, by adding the chemicals into the water while stirring. Check pH, adjust to pH 6.8 if necessary, and fill up to final volume with distilled water. Can be stored at RT for at least 3 months.

##### McIlvain (1921) (phosphate-citrate) stock A (1 l)

Dissolve 21 g of citric acid monohydrate in 800 ml of distilled water. Add up to final volume with distilled water. Can be stored at RT for at least 3 months.

##### McIlvain (1921) (phosphate-citrate) stock B (1 l)

Dissolve 35.6 g of disodium phosphate dihydrate in 800 ml of distilled water. Add up to final volume with distilled water. Can be stored at RT for at least 3 months.

##### McIlvain (1921) (phosphate-citrate) buffer pH 4 (1 l)

Mix 614 ml of stock A with 386 ml of stock B. Check pH and if necessary, adjust to pH 4 by adding the respective solution.

##### 50% (w/v) potassium disulfite solution (1 l)

Dissolve 500 g of potassium disulfite in 800 ml of distilled water. Add the potassium disulfite to the water while stirring. Heat the solution up to 70–80 °C and keep stirring until everything is dissolved. Do these steps in the fume hood! Potassium disulfite has a very strong smell. Add up to final volume with distilled water. Potassium disulfite can be purchased as crystals or powder. Powder tends to dissolve faster. If the solution has been stored for a while, crystals will precipitate. Therefore, always reheat stir the solution until the precipitate is dissolved again and filter before use. The solution can be re-used many times. Can be stored at RT for at least 3 months.

##### 1% Methylene blue stock solution (100 ml)

Dissolve 0.1 g of methylene blue in 80 ml of McIlvain buffer pH 4, by stirring overnight. Add up to final volume with McIlvain buffer pH 4. The solution can be stored at 4 °C in the dark for many months. If you observe fungal growth, try to add 1–2 crystals of thymol. This has generally not been necessary for us.

##### 1% Neutral red stock solution (100 ml)

Dissolve 0.1 g of neutral red in 80 ml of McIlvain buffer pH 4, by stirring overnight. Add up to final volume with McIlvain buffer pH 4. The solution can be stored at 4 °C in the dark for many months. If you observe fungal growth, try to add 1–2 crystals of thymol. This has generally not been necessary for us.

##### 2% PBST (1 l)

Add 100 ml of 10 × PBS and 2 ml of Triton X-100–700 ml distilled water. Triton X-100 is very viscous. Cut off the tip of a 1 ml micropipette tip for better pipetting and eject the pipette tip into the solution while stirring until all the Triton X-100 is dissolved. Check pH, adjust to pH 7.4 if necessary, and fill up to final volume with distilled water.

##### PBS + 10 mm SDS (SWITCH-OFF) (Murray et al. 2015) buffer (1 l)

Add 100 ml of 10 × PBS to 700 ml distilled water. Dissolve 2.9 g sodium dodecyl sulphate (SDS) under stirring. Check pH, adjust to pH 7.4 if necessary, and fill up to final volume with distilled water.

##### Methylene blue working solution (1 l)

Dilute 0.1% methylene blue stock solution 1:100, by adding 10 ml of stock solution to 990 ml McIlvain buffer pH 4.

##### Neutral red working solution (1 l)

Dilute 0.1% neutral red stock solution 1:100, by adding 10 ml of stock solution to 990 ml McIlvain buffer pH 4.

##### 4% agar solution (1 l)

Dissolve 40 g agar in 800 ml distilled water by heating it up under stirring. Cover the container with aluminium foil to prevent evaporation and boil while stirring until the solution becomes clear. Fill up to final volume with distilled water and let cool down until hand warm. Optional: If agar is to be stored long term, add a few crystals of thymol to the solution and stir until dissolved. The solidified agar can be stored at 4 °C for months and melted in a microwave or water bath.

#### Human material

Brain tissue samples were taken from four different human body donors (Occipital lobe 1: 82 year old male with no known neuropathological diseases; Occipital lobe 2: 101 year old female with no known neuropathological diseases; Occipital lobe 3: 92 years old female with diagnosed dementia; Occipital lobe 4: 98 year old male with no known neuropathological diseases; post-mortem accumulation of blood in the occipital pole) of the body donation program of the Department of Anatomy and Embryology, Maastricht University. The tissue donors gave their informed and written consent to the donation of their body for teaching and research purposes as regulated by the Dutch law for the use of human remains for scientific research and education (“Wet op de Lijkbezorging”). Accordingly, a handwritten and signed codicil from the donor posed when still alive and well, is kept at the Department of Anatomy and Embryology Faculty of Health, Medicine and Life Sciences, Maastricht University, Maastricht, The Netherlands. All experiments have been approved by the ERCPN ethics board of the Faculty for Psychology and Neuroscience at Maastricht University, Maastricht, The Netherlands. The authors hereby confirm that every effort was made to comply with all local and international ethical guidelines and laws concerning the use of human cadaveric donors in anatomical research.

### Methods

#### Human brain tissue fixation protocol

All body donors underwent the same standardized fixation routine at the Department of Anatomy and Embryology Faculty of Health, Medicine and Life Sciences, Maastricht University, Maastricht, The Netherlands:

1. Brains are first fixed in situ by full body perfusion via the femoral artery. Under a pressure of 0.2 bar the body was perfused by 10 l fixation fluid (1.8 vol% formaldehyde, 20% ethanol, 8.4% glycerin in water) within 1.5–2 h.
2. Thereafter the body is preserved for at least 4 weeks for post-fixation submersed in the same fluid.
3. Subsequently, brains are recovered by calvarian dissection and stored in 4% paraformaldehyde in 0.1 M phosphate buffered saline (PBS) for 14–30 months until use.

#### Preparation of human brain slices

**Fig. 1:**
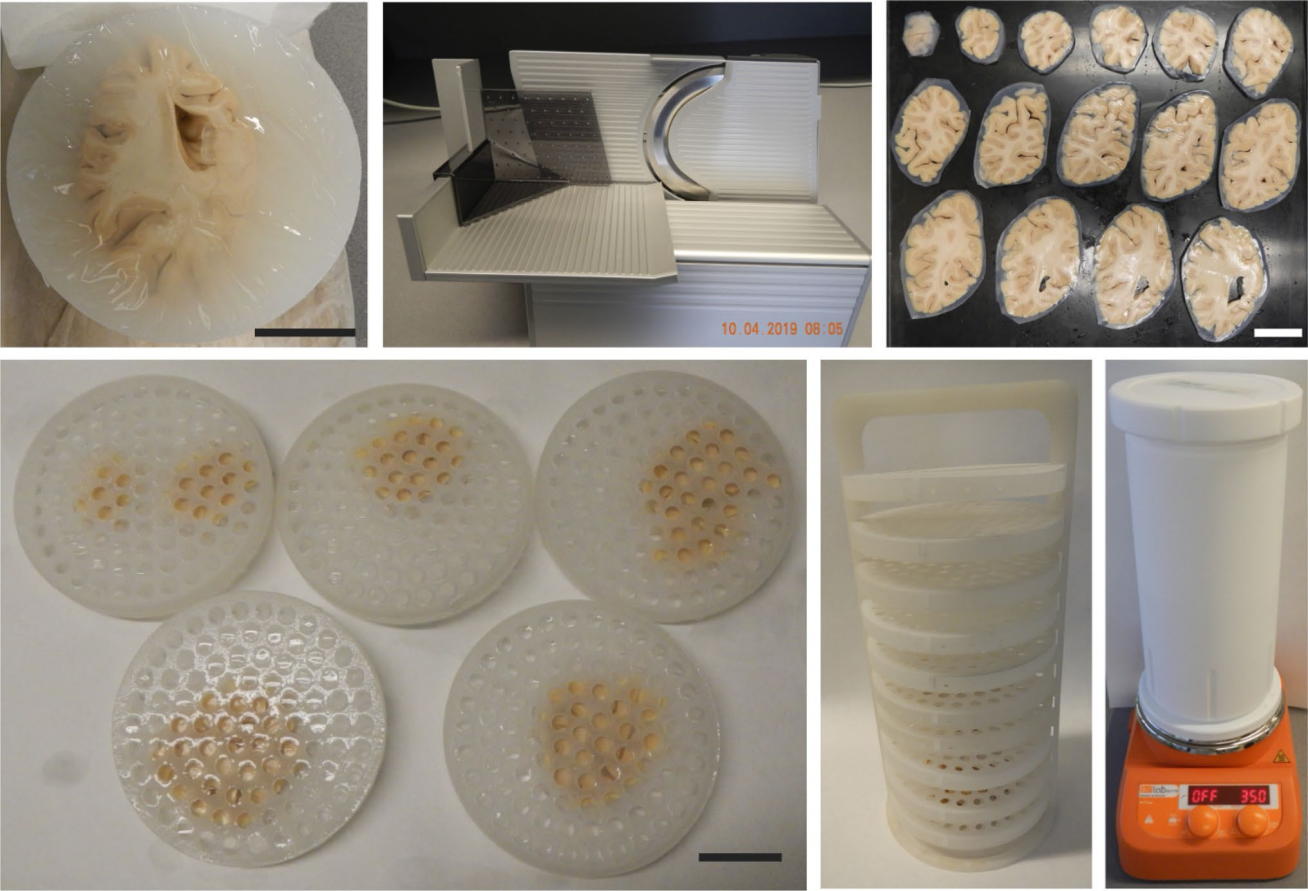
Overview of the tissue-processing pipeline. Whole occipital lobes were embedded in 4% agar (**a**) and cut into approx. 3 mm thick coronal slices on a commercial rotation slicer (**b**). The surrounding agar ring was trimmed with a scalpel (**c**) to better accommodate the samples in the sample holder discs (**d**). The SLS printed tower (**e**) can fit up to 10 sample holder discs with a 10 cm inner diameter and inner height of 5 mm. This amounts to a theoretical maximal tissue volume of over 390 ml. The PTFE container (**f**) can accommodate a volume of approx. 2 l of staining and clearing chemicals with a fully loaded tower, guaranteeing a sufficient volume of staining and clearing solutions for the large tissue volume, while fitting of a compact magnet stirrer. Scale bar: 3 cm, respectively

**Note:** By letting the 4% agar solution cool down, most of the bubbles in the solution should have risen to the surface. If bubbles in the agar are a problem, because they cause fracturing of the agar block during the slicing, the agar can be degassed with a vacuum pump before use. We usually do not find this step necessary.

Each of the occipital lobes is cut into approx. 3 mm thick coronal slices on a conventional rotation meat slicer (ritterwerk GmbH, Gröbenzell, Germany). To keep the brain tissue stabilized during slicing, the occipital lobes are embedded in 4% agar (see Fig. 1 a and supplementary Fig. 1).

1. For this, the tissue is placed with the flat, cut surface of the anterior side in a plastic pipe with 12 cm diameter purchased from a local hardware store.
2. The pipe is in turn fixed with putty on a plastic plate.
3. Wait until the agar is hand-warm slowly fill the pipe until the tissue is completely covered.
4. Once filled with agar, the pipe is surrounded with crushed ice to accelerate solidification of the agar and can be stored in a cold room as well to further accelerate the process.
5. The agar ring around the coronal slices is trimmed, but not completely removed unless it detached by itself during the later processing steps, as it serves to keep unattached gyri in place.
6. If desired, block the tissue into smaller pieces containing the region of interest.

For this demonstration, of occipital lobe 1 and 2, the first 6 whole slices (posterior to anterior) where processed with MASH (Hildebrand et al. 2019) and labelled for cytoarchitecture (hereafter called cytoMASH). In addition, for occipital lobe 3, smaller samples of 2–3 cm lateral size were blocked from slices, cleared with MASH and labelled for both cyto-and angioarchitecture (angioMASH) (Hildebrand et al. 2024).

#### 3D printed sample holders

Designs for 3D printable sample holders were created either in SOLIDWORKS (Dassault Systèmes SolidWorks Corporation, Waltham, US) or in FreeCAD (version: 0.18; https://www.freecadweb.org). For the 3D printing, two different printing techniques were tested: Selective Laser Sintering (SLS) provided by a 3D printing company and Fused Deposition Molding (FDM)/ Fused Filament Fabrication (FFF).

SLS printed sample holders: A custom-made sample holder (see supplementary files 1–3) optimized for coronal lobe slices of up to 5 mm thickness were produced from polypropylene (PP), after initial tests confirmed that PP is chemically resistant to dichloromethane (DCM), an organic solvent that can corrode and even dissolve many plastics such as polystyrene or polylactic acid. We noted that the material undergoes slight expansion during the DCM incubations and reverts to its original size after DCM is completely evaporated, but to the best of our awareness, this had no negative impact on the procedure. A first prototype (Fig. 1d-f and supplementary Fig. 1) was produced via SLS by the 3D printing company Materialise NV (Leuven, Belgium).

**Fig. 2:**
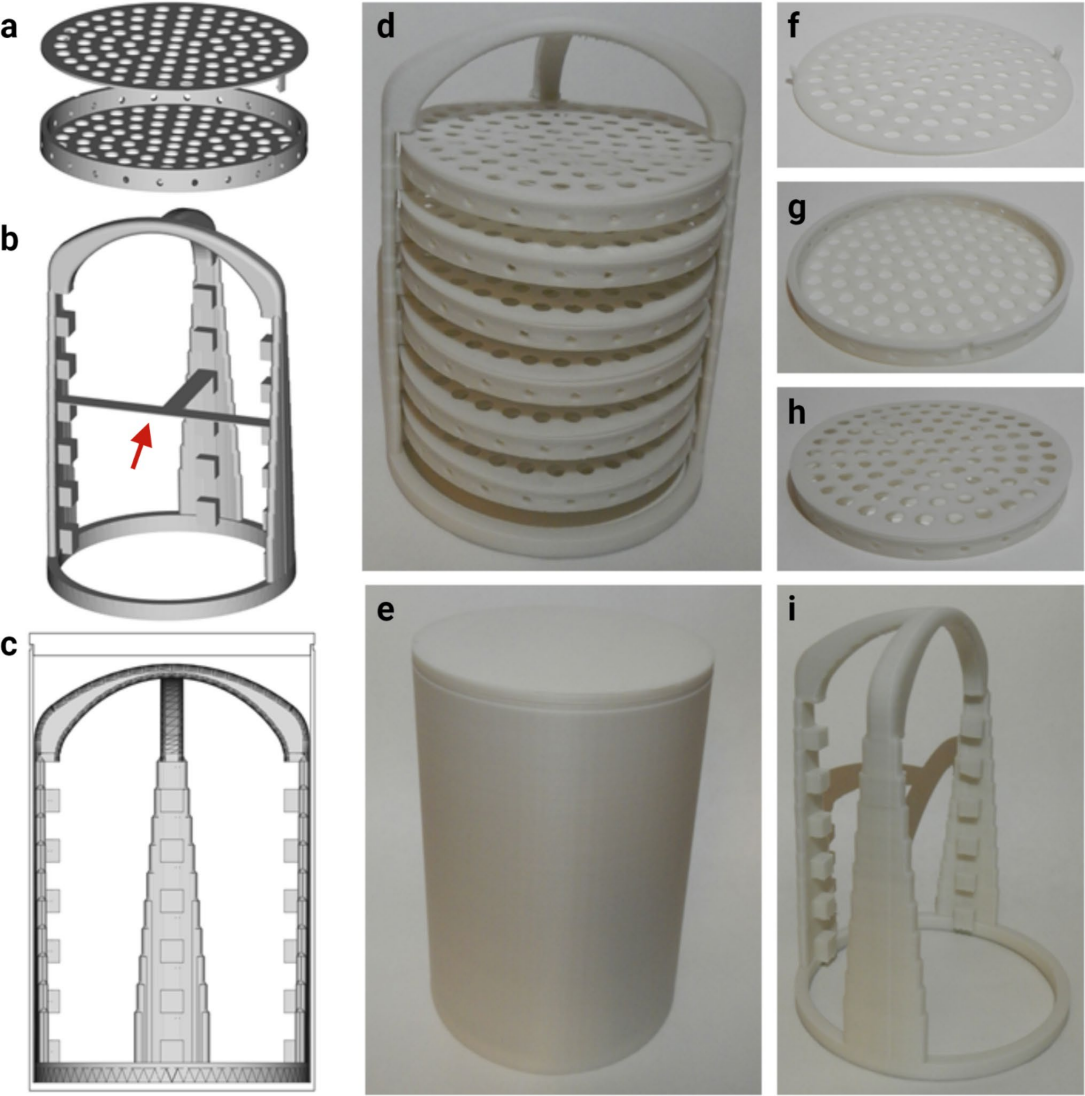
Modified clearing tower version for FDM/FFF printing. **a** The dovetails on the lids and corresponding groves on the sample holder discs were modified to remove 90⁰ angles for better printability. **b** To prevent the tower poles from swinging through the movement of the nozzle during printing, additional support elements (red arrow) were introduced, and the tower pole width was increased as well. This together with the third pole added to the design also gives more support to the sample holder discs. **c** Tower inside the custom-made clearing container. **d**—**i** Final prototype printed with PP filament

**Note:** PP filament has a tendency to warp (bend upwards) during the printing. This is especially challenging for larger prints such as these. If this proves the case, try to add more adhesive. Additionally, try to lower the nozzle tip as low as possible onto the plate, but take care not to touch the plate. In our version of the Ender 3D printer, this can be adjusted during the printing of the first layer with the “z baby step” option. Check if your printer has a similar option. If the soft PP filament is not extruded correctly, adjust the flow rate of the extruder. For this, measure with a ruler if a certain length (e.g., 5 cm) that is supposed to be extruded by the extruder stepper motors is actually of that length. Make sure that your printer stands in a stable environment without drafts or air flow, if the printer has an open design and no surrounding chamber.

FDM/FFF printed sample holders: Following initial tests, an adjusted design was developed to provide more stability for the sample holder discs within tower, while adjusting the design to be printable with FDM/FFF printers at the same time (see supplementary files 4–8). These modified versions were printed on an Ender 3 Pro printer (Creality, Shenzhen, China) as follows:

1. A PP plate used as the building platform of the printer is coated with Magigoo PP adhesive (Thought3D Ltd, Malta).
2. The Fiberology PP filament (Fiberlab S.A., Brzezie, Poland) is printed at 230 ⁰C nozzle temperature and 100 ⁰C bed temperature with a 0.4 brass nozzle at 0.1 mm layer height and 0.35 mm/s. Set the filling density of the structure to 50% and print support structures as a grid with 25% filling to ensure good quality of the print.
3. Support structures, including the support connection at about half the tower height (see red arrow Fig. 2b) are removed with a crafting knife, scalpel, or similar, and wire cutters.

#### Optical tissue clearing and labelling of human brain samples

Depending on the sample size, the optical tissue clearing can be carried out in various ways. For very large samples such as whole coronal sections of Occipital lobe 1 and 2, the complete procedure is carried out in the custom-made 3D printed sample holder (see Sect. "McIlvain (McIlvaine, 1921) (phosphate-citrate) stock B (1 l)" and Fig. 1). The SLS sample holder is placed into a polytetrafluorethylen (PTFE) container (Thermo Fisher Scientific, Waltham, Massachusetts, U.S.). FDM/FFF printed sample holders are placed in 3D printed PP containers. For all steps, ~ 1–1.2 l of every solution was used, which was sufficient to cover all 6 samples of each lobe. When all the 10 discs of the larger SLS sample holder contain tissue, about twice that volume (~ 2 l) would be needed. The container was kept on a magnet stirrer throughout the incubations at 200 rpm. For smaller samples of a lateral size of 2–3 cm, 6 well cell culture plates can be used as well for the clearing and labelling procedure. This is a convenient solution for fast piloting of smaller sample sizes, as the materials are readily available in most laboratories and the required volume per well is small (~ 5–6 ml are sufficient to cover 5 mm think samples). 25 ml glass pipettes are used to empty wells and replace the solutions. Importantly, cell culture plates are made of polystyrene and are not compatible with the delipidation solution (see below). Therefore, tissue samples have to be transferred to 50 ml test tubes made of PP. For very large sample sizes, it would still be recommended to use the 3D printed sample holders e.g., for better within-batch consistency. As several 2–3 cm samples can be placed in the same disc, this would also increase the throughput considerably.

Samples were processed as described previously(Hildebrand et al. 2024, 2019; Schueth et al. 2023). A detailed step-by-step description of the processing pipeline is provided below with notes and adjustments that deviate slightly from the originally published method.

#### cytoMASH protocol

1. Day 1: Samples are dehydrated 1 h each in 20, 40, 60, 80, 100% methanol (MeOH) at room temperature (RT) and 1 h in 100% MeOH at 4 ⁰C. Samples are then bleached overnight in freshly prepared, chilled 5% H_2_O_2_ in MeOH (v/v) at 4 ⁰C. For steps at 4 °C, the samples are stored in a cold room. For all steps throughout the protocol, the samples are either placed on an orbital shaker at 50–60 rpm (for smaller occipital lobe 3 samples in 6 well cell culture plates) or a magnetic stirrer at 200 rpm (for occipital lobe 1 and 2 samples in the 3D printed sample holders).
2. Day 2: The samples are rehydrated in 60, 40, 20% MeOH at RT for 1h, respectively. This was followed by permeabilization for 2 × 1 h in 0.2% PBST (phosphate buffered saline + 0.2% (v/v) Triton x-100 adjusted to pH 7.4) and a second bleaching step in 50% (w/v) aqueous potassium disulfite solution for 1h at RT. Samples are thoroughly rinsed 5 × in distilled water and washed for 1h in distilled water at RT. Then, samples were stained in 0.001% neutral red or 0.001% methylene blue in McIlvain buffer (McIlvain, T. C. 1921) (w/v) at pH 4 for 5 days. To make 1l of McIlvain buffer (phosphate-citrate buffer) at pH 4 mix 386 ml of a 0.2 M disodium hydrogen phosphate solution with 614 ml of a 0.1 M citric acid solution. Check pH and if necessary, adjust by adding the respective solution.
3. Day 5: Samples are flipped after half the incubation time.
4. Day 8: Samples are washed again 2 × 1 h in McIlvain at pH 4 and dehydrated 1 h each in 20, 40, 60, 80, 2 × 100% MeOH/H_2_O (v/v). For delipidation, the samples are placed in 66% DCM/33% MeOH overnight.
5. Day 9: The samples are washed 2 × 100% DCM. Finally, they are immersed in ethyl cinnamate (ECi) overnight.
6. Day 10: ECi is exchanged the next morning and samples are optically checked for transparency. The samples should be ready for microscopic imaging now.

**Note:** The second bleaching step with potassium disulfite at point 4.1.2. was originally introduced to improve the cytoarchitecture staining in archival tissue (Böck, P. 1979). However, we have observed that most tissues bleach visibly in this solution and obtain a creamy-white colour (see supplementary Fig. 3 as well as top right views of the samples in supplementary Fig. 4–15). We also observed that this step improves the contrast of the fluorescent cytoarchitecture staining (see supplementary Fig. 4 and 5 in (Hildebrand et al. 2019)). One potential drawback of this solution is the low pH and depending on the desired label, the pH must be carefully adjusted afterwards. At point 4.1.3., check if the staining solution still has visible colour, or if the dye is depleted. If the dye is depleted, replace the staining solution. At 4.1.4., if 6 well cell culture plates have been used so far, place the samples on 50 ml PP test tubes before this step and use them for all following steps! If the centre of the samples or white matter regions still look murky or opaque at 4.1.6., leave in the ECi for another day. All steps involving MeOH and/or DCM should be carried out in a fume hood. Make sure to wear chemically resistant gloves when handling DCM, as it is a suspected carcinogen. The potassium disulfite solution has a very strong and pungent smell and should also be prepared in a fume hood.

#### angioMASH with cytoMASH counterstaining protocol

1. Day 1: Samples are dehydrated 1 h each in 20, 40, 60, 80, 2 × 100% MeOH/H_2_O (v/v). For better label penetration of the lectin, the delipidation is performed before the staining in this version. The samples are placed in 66% DCM/33% MeOH overnight.
2. Day 2: The samples are washed 2 × 100% DCM. Samples are then bleached in freshly prepared, chilled 5% H_2_O_2_ in MeOH (v/v) at 4 ⁰C as described above for 8 h. The samples are stored overnight in 80% MeOH/H_2_O (v/v), although no negative effects have been observed when samples were left in the bleaching solution until the following day.
3. Day 3: The samples are rehydrated in 60, 40, 20% MeOH at RT for 1 h respectively. Since the potassium disulfite solution lowers the pH of the tissue, this step is done before the permeabilization in PBST in this version of the protocol. A low pH can have a negative effect of the lectin staining, although the label is generally very robust to smaller pH changes. Samples are bleached in 50% (w/v) aqueous potassium disulfite solution for 1 h at RT. Samples are thoroughly rinsed 5 × in distilled water and washed for 1h in distilled water at RT. This was followed by permeabilization for 2 × 1 h in 0.2% PBST. The samples are then equilibrated in PBS + 10 mM SDS (SWITCH-OFF buffer)(Murray et al. 2015) overnight at RT.
4. Day 4: Samples are stained in DyLight® 649 conjugated *Lycopersicon esculentum* lectin (LEL; 1:100–1:200 dilution or 5–10 µg/ml) in SWITCH-OFF buffer for 5 days.
5. Day 7: Samples are flipped after half the incubation time.
6. Day 10: Samples are washed 5 × 1 h in PBST, followed by overnight incubation in PBST at RT (SWITCH-ON step).
7. Day 11: Samples are washed 3 × 1 h in PBST and 3 × 1 h in McIlvain buffer at pH 4. Then, samples are stained in 0.001% neutral red in McIlvain buffer (w/v) at pH 4 for 5 days.
8. Day 14: Samples are flipped after half the incubation time.
9. Day 17: Samples are washed again 2 × 1 h in McIlvain at pH 4 and dehydrated 1 h each in 20, 40, 60, 80, 2 × 100% MeOH/H_2_O (v/v). Samples are placed in 100% DCM for 1 h for complete dehydration.
10. Day 18: ECi is exchanged the next morning and samples are optically checked for transparency. The samples should be ready for microscopic imaging now.

**Note:** At point 4.2.1. and 4.2.9., if 6 well cell culture plates have been used so far, place the samples on 50 ml PP test tubes before this step and use them for the following three steps! At 4.2.2., if the samples were placed in 50 ml tubes until now, they can be put back into 6 well cell culture plates for the 80% MeOH and following steps. This is not necessary but more economical, because of the smaller volumes required. At 4.2.5. and 4.2.8., check if the staining solution still has visible colour, or if the dye is depleted. If the dye is depleted, replace the staining solution. If the centre of the samples or white matter regions still look murky or opaque at 4.2.10., leave in fresh ECi for another day. All steps involving MeOH and/or DCM should be carried out in a fume hood. Make sure to wear chemically resistant gloves when handling DCM, as it is a suspected carcinogen. The potassium disulfite solution has a very strong and pungent smell and should also be prepared in a fume hood.

#### Oblique light-sheet fluorescence microscopy

To showcase our tissue-processing pipeline, samples were imaged on one of two systems: Whole occipital lobe slices stained for cytoarchitecture were imaged on the ct-dSPIM (cleared tissue dual-view Selective Plane Illumination Microscopy) prototype set-up (Schueth et al. 2023) (Applied Scientific Instrumentation, Inc., Eugene, US) and smaller occipital lobe blocks stained for both angio- and cytoarchitecture were imaged on the QuVi SPIM (LUXENDO GmbH, Heidelberg, Germany).

#### Sample mounting on the ct-dSPIM

The samples are mounted in a custom-made imaging chamber (see supplementary file 9). For details on the imaging chamber, we refer the reader to the original publication(Schueth et al. 2023).

1. Samples are taken out of ECi and blotted dry with paper towel. Leave samples wrapped in paper towels for a few minutes.
2. Dry samples are glued onto the glass surface of the ct-dSPIM imaging chamber. Take care to only have adhesive in the centre under the sample. Do not use so much adhesive that it will squelch around the sides as this may impede light penetration and data quality.
3. The sample chamber is filled with ECi until the sample is barely covered with liquid.
4. Install the chamber in the ct-dSPIM mechanical stage and fill up to immerse the objective lenses fully.

**Note:** As adhesive, several glues have been used successfully by our group over the years. We have employed cyanoacrylate super glue (Gorilla Glue, Cincinnati, Ohio, USA), a hot glue gun (Bison International B.V., Goes, the Netherlands), and fast-curing sanitary silicone sealant (Gorilla Glue, Cincinnati, Ohio, USA and Bison International B.V., Goes, the Netherlands). All adhesives were obtained from a local hardware store. Super glue cures very fast and is well suited for smaller pieces, however we have observed that in some cases, the low viscosity glue can penetrate the tissue and reduce the transparency. The hot glue gun worked well for shorter imaging session, but care must be taken not to use too much, so that the glue does not enter gyri or forms a ring around the outside of the sample. This impedes light penetration. In addition, samples sometimes detached during very long imaging sessions (overnight). The silicone sealant takes the longest time to cure, so samples need to be mounted well in advance before imaging. We have observed no negative effects of keeping the transparent, dehydrated samples dry for the time it takes for the silicone to cure (30–120 min depending on brand). No negative results on tissue transparency have been observed with this method.

#### Image acquisition on the ct-dSPIM

Occipital lobe 1 and 2 samples were imaged on the ct-dSPIM. The set-up is equipped with multi-immersion detection objectives (Applied Scientific Instrumentation, Inc., Eugene, US/Special Optics, Denville, US), suitable for a refractive index range from 1.33 to 1.56 and with a working distance (WD) of 12 mm. Numerical aperture (NA) and effective focal length (EFL) vary with refractive index, but for ECi the NA is ~ 0.43 and EFL ~ 11.6 mm.

For excitation of the neutral red dye, an OBIS LS 552 nm 40 mW laser line (Coherent Inc., Santa Clara, US) is used. Samples were imaged with 10 ms exposure time at the lowest laser intensity setting with 4 × 4 binning (512 × 512 pixels) and a step size of 23.2 µm. This step size allows for fast, mesoscopic overview acquisitions of whole occipital lobe slices.

#### Sample mounting on the QuVi SPIM

1. Samples are taken out of ECi and blotted try with paper towel. Leave samples wrapped in paper towels for a few minutes.
2. Dry samples are glued onto the surface of PP chambers, 3D printed with FDM/FFF printing (see supplementary file 10). As adhesive, UV curable BONDIC® glue was used: A small drop of the glue was placed on the chamber and the sample gently placed on top. The glue was cured by shining the included UV LED on the periphery of the sample at surface height. Take care to only have adhesive in the centre under the sample. Do not use so much adhesive that it will squelch around the sides as this may impede light penetration and data quality.
3. The sample chamber is filled with ECi until the sample is barely covered with liquid.
4. Install the chamber in the QuVi SPIM and fill up to immerse the objective lenses fully.

#### Image acquisition on the QuVi SPIM

Occipital lobe 3 samples were imaged on the QuVi SPIM. The QuVi SPIM uses the same objectives as the ct-dSPIM, described in 5.2. The data were acquired with an exposure time of 40 ms and an initial voxel size of 0.363 µm × 0.363 µm × 2.9 µm. The far-red LEL label was excited with an OBIS LX 642 nm 60 mW laser line at 12% intensity. A 656 nm long-pass emission filter was used. The neutral red label was excited with an Obis LX 561 nm 40 mW laser line at 6% intensity. A BP 580–627 nm band-pass filter was used on the emission side. The data was acquired by a LUXENDO employee during a loan of the system.

#### Data processing

For the creation of all figures, BioRender was used (https://www.biorender.com).

#### ct-dSPIM data processing

1. To obtain an isotropic mesoscale dataset of 16.4 µm, as shown for occlobe 1 and 2 samples, images are further downsampled 16 × in plane (32 × 32 pixels), to match the step size of the microscope after deskewing.
2. Downsampling is done in FIJI(Schindelin et al. 2012) with the inbuilt “Scale” function (Image > Scale).
3. The downsampled.tiff stacks are then imported and resaved as.h5 file with the “Automated Loader” option in BigStitcher plugin(Hörl et al. 2019) for FIJI.
4. The stacks are arranged into the correct mosaic via “Arrange Views” > “Move Tiles to Regular Grid”. This is done by visually checking for the correct orientation in all three dimensions with the viewer.
5. The correctly arranged image stacks are deskewed with “Arrange views” > “(De)Skew Images” either at 45⁰ or −45⁰. This will depend on the side of the microscope that was used for the image acquisition and can again be checked visually using the viewer. If the wrong angle was selected, this can be corrected with “Calibration/Transformation” > “Remove Transformation” > “Remove Latest/Newest Transformation”
6. Stitching of the datasets is performed in BigStitcher following the “step-by-step” stitching option. For this the default options usually give good results. First “Calculate Pairwise Shifts” > “Phase Correlation” and do not select any downsampling in the next window as the data is already downsampled. Then inspect the results with “Verify/Filter Pairwise Links” > “Interactive Link Explorer”. Any grossly wrong links can be manually removed after visual inspection in the viewer. With the downsampled data, the cross-correlation between pairs is usually very high. Therefore, the “filter by correlation coefficient min R” value can often be increased beyond the default 0.7 value. Finally select “Optimize Globally and Apply Shifts” > “Simple Mode” and run with the default option selected in the next window.
7. For export and fusion, select “Image Fusion”. In the pop-up window select from top to bottom: The default bounding box for the entire volume, no further downsampling (value 1), “Linear Interpolation”, “Avg, Blending”, “16-bit unsigned integer”, “Disable Non-Rigid”, “All views together” and “Display using ImageJ” option.
8. Save the newly opened dataset as a.tiff stack via FIJI (“Safe As” > “Tiff”).
9. This new volume is resliced (“Image” > “Stacks” > “Reslice [/]”) in FIJI to show the coronal plane of the tissue slice (YZ plane of the image volume).

#### QuVi SPIM data processing

The datasets of occipital lobe 3 samples were deskewed, downsampled to 2.9 µm isotropic voxel size, and stitched with the proprietary LuxProcessor, part of the LuxBundle software of LUXENDO GmbH (Heidelberg, Germany), immediately after raw data acquisition by a LUXENDO employee. An additional.tiff stack was generated with a further downsampled isotropic voxel size of 8 µm with the LuxBundle software. For visualization in Fig. 5, and Supplementary Movie 1, the data was equalized using a strategy similar to the one implemented in ClearMap toolbox(Kirst et al. 2020). Briefly, the image was divided by a normalization image, which captures the slow decay of intensity caused by light scattering, calculated as the local median intensity. To avoid the contamination of the normalization image by the sharp intensity difference between sample and imaging-medium, calculation of the normalization image was restricted to the sample. For this purpose, a subset of 2D images were selected along the z-axis, and manually segmented into brain (foreground) and background using the napari-nD-annotator. In the normalization image, regions belonging to the background were filled with the nearest value in the foreground using a euclidean distance transform. For computational speed, the local median was calculated at a lower resolution than the original image and then up-sampled to the original image. Finally, images were rendered in 3D using the Amira software (Thermo Fisher Scientific Inc., Waltham, Massachusetts, U.S.A.).

## Results

Our high-throughput version of the MASH protocol (Fig. 1) allows for rapid optical clearing and cytoarchitecture labelling in very large tissue volumes. Large parts of e.g., human occipital lobes can be histologically processed and ready for microscopic imaging within 10 days. In the SLS printed prototype (Fig. 1d and e; supplementary Fig. 2), a maximum tissue volume of approx. 390 ml (in slices with a diameter of 10 cm) can be processed in a single round of clearing. This pipeline considerably extends the current clearing and labelling capacities, while requiring mostly standard lab equipment and little additional, costly hardware.

To further reduce the cost of the pipeline, we replaced the most expensive hardware item, the SLS printed clearing tower and sample holders. The new design can be reliably printed with PP filament of standard FDM/FFF desktop printers (Fig. 2), making the whole set-up for the histological pipeline yet more economical. The FDM/FFF prints have a lower resolution, but the printing quality is sufficient for all the parts at this large scale. The slightly smaller filament printed tower fits 6 sample holder discs corresponding to a maximum tissue volume of 235 ml.

**Fig. 3:**
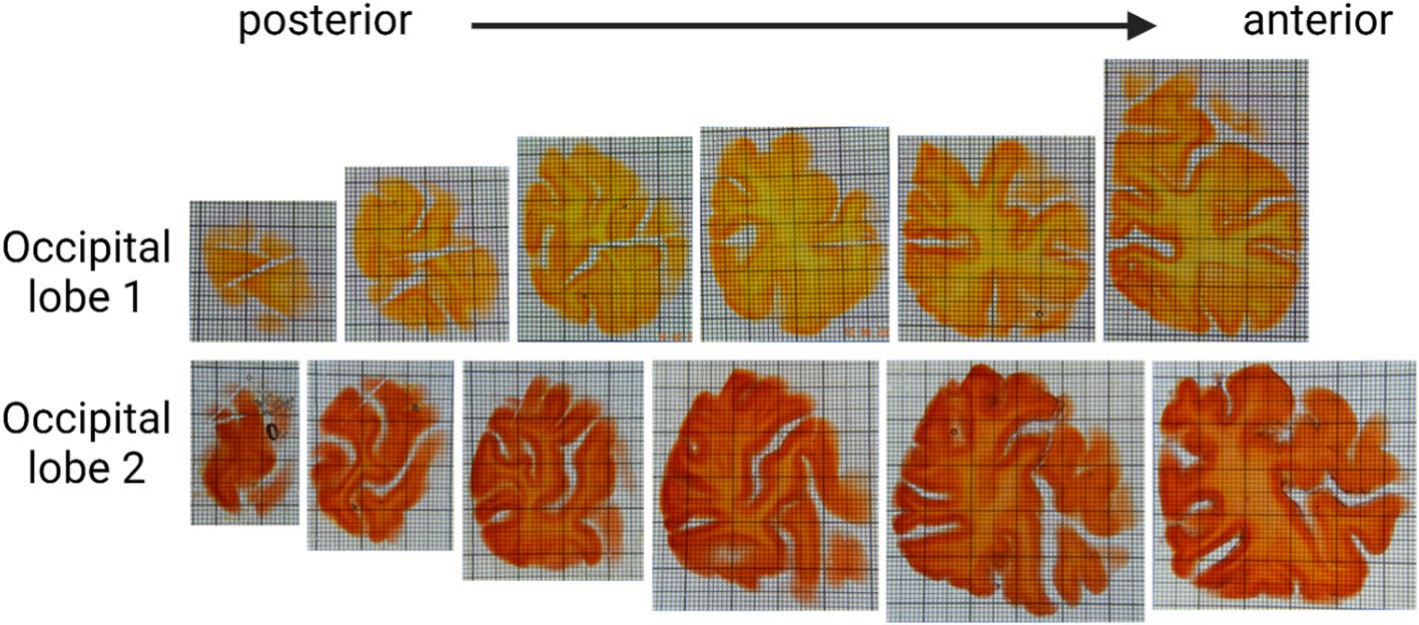
High-throughput OTC of two human occipital lobes. All processed coronal slices of occipital lobe 1 (top) and occipital lobe 2 (bottom) arranged with the posterior pole towards the left side of the image and the anterior most sample towards the right. All panels are adjusted to the same scale (grid size: 1 × 1 mm smallest squares; 10 × 10 mm boldly lined squares)

To provide a proof of principle, we processed the posterior portions of two human occipital lobes (Fig. 3 and supplementary Fig. 4–15). For this, six consecutive 3 mm thick slices starting at the occipital pole were cleared and labelled in our new high-throughput set-up. Even the dense, central white matter in these large samples became highly transparent. Within each batch, the labelling appeared homogeneous independent of the sample size.

**Fig. 4:**
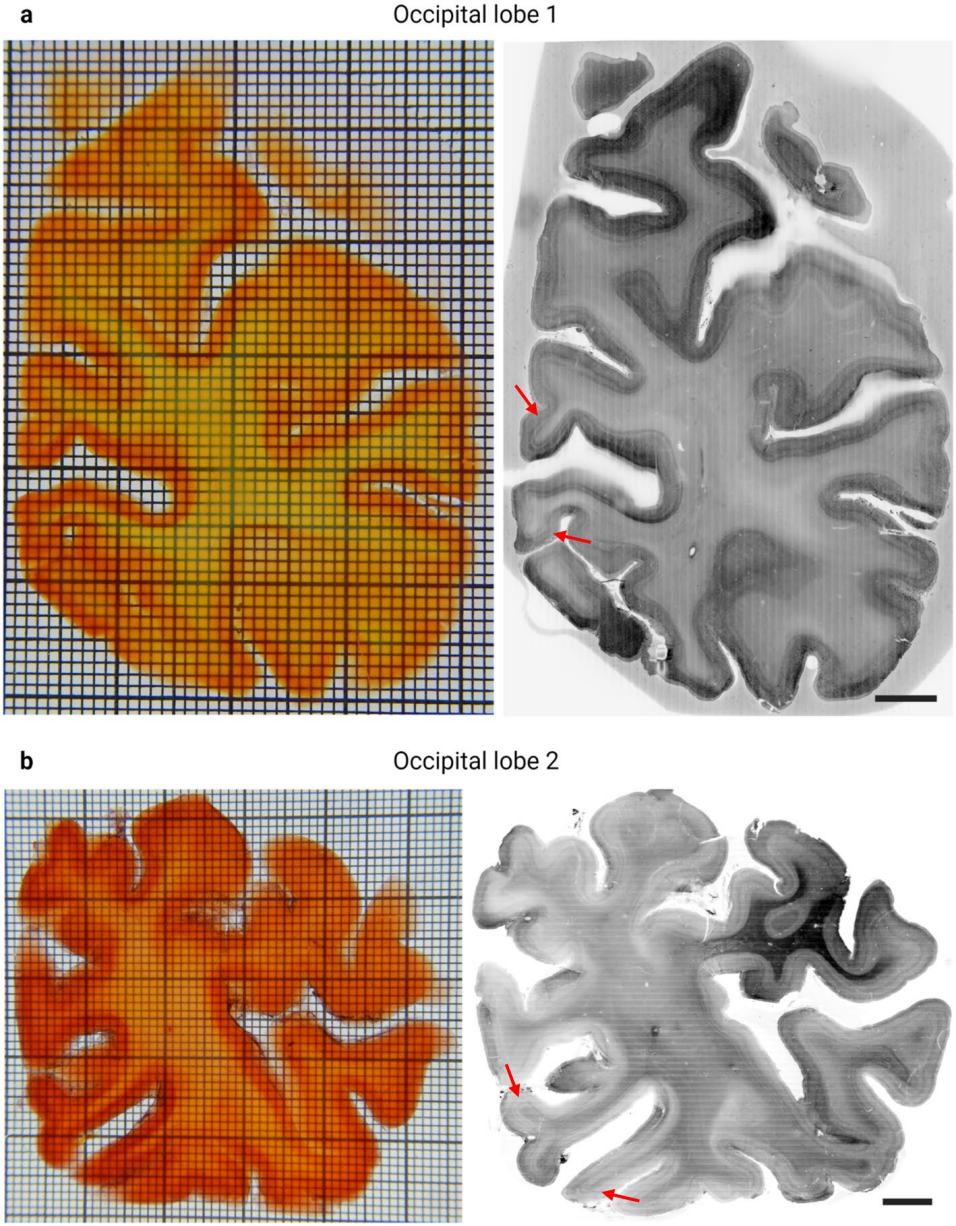
Mesoscopic overview acquisitions of anterior most occipital lobe slices. Comparison between the macroscopical images of the cleared samples (right side) and the stitched, resliced image volumes in inverted greyscale (left side) of the two largest slices of occipital lobe 1 (**a**) and occipital lobe 2 (**b**). Both panels of the microscopy volumes show MIPs of approx. 50 µm showing the largest extend of the samples. Anatomical landmarks such as the V1/V2 border can be distinguished even at mesoscopic resolution (arrows). Scale bars: 5 mm, respectively; grid size: 1 × 1 mm smallest squares; 10 × 10 mm boldly lined squares

To assess the labelling quality of the samples, mesoscopic overview scans of the largest slice of occipital lobes 1 and 2 were acquired (Fig. 4). At mesoscopic resolution of 16.4 µm isotropic, anatomical landmarks such as the tentative V1/V2 border are already visible (Fig. 4 read arrows), as are several cytoarchitectonic layers. As expected, the small molecule label penetrates the entire depth of the large samples after 5 days of passive incubation (see supplementary Figs. 16 and 17; supplementary video 1 and 2).

**Fig. 5:**
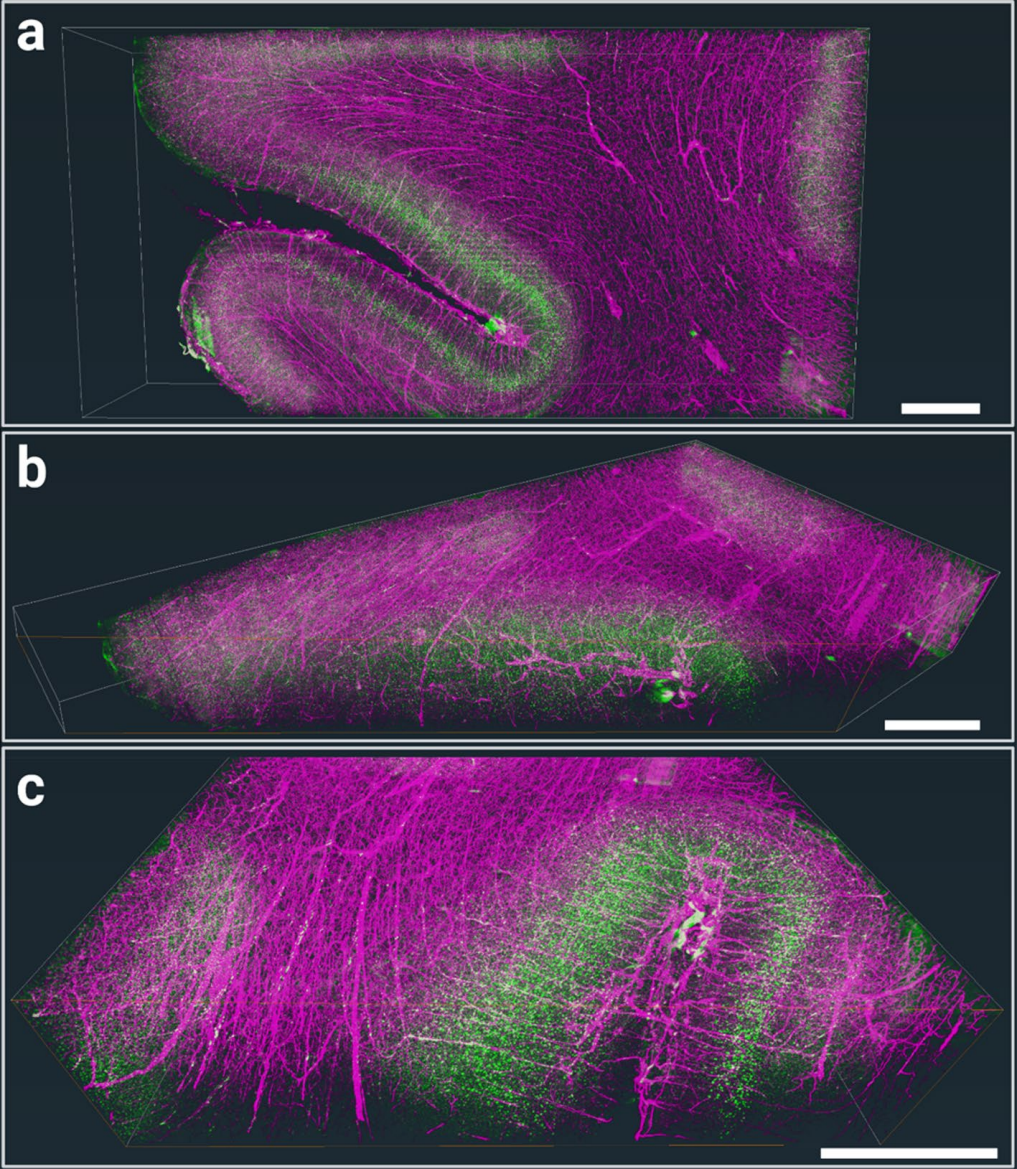
Combined angio- and cytoMASH OTC and staining on human occipital lobe blocks. **a** Top view onto the surface (coronal orientation) of the 3 mm thick piece. Note that even at mesoscopic resolution of 8 × 8 × 8 µm voxel size, conspicuously large cells (green) in infragranular layers are well visible. **b** Sample with an oblique clipping plane into the cortex showing the dense vasculature (magenta) in the gyral crown. **c** Zoom-in to an orthogonal clipping plane roughly perpendicular to the larger gyrus. The mesoscopic diving vasculature that runs perpendicular to the pial surface is well visible. Scale bar: 3 mm, respectively

Even the larger lectin molecules penetrate well into thick samples (Fig. 5; supplementary Fig. 18; supplementary video 3), although a falloff in signal intensity due to increased light scattering with imaging depth is visible. Even at a mesoscopic resolution of 8 µm isotropic voxel size, some angio- and cytoarchitectonic feature are well visible, such as a conspicuous layer of large cells in the deeper half of the cortex and diving blood vessels running perpendicular to the cell layers. These radial vessels are especially pronounced in the superficial half of the cortex (Fig. 5c), while the orientation appears predominantly tangential (parallel to the cell layers) towards both, the while matter as well as on the pial surface.

## Discussion

We demonstrate an updated version of MASH for the cost-efficient, robust, and routine application of OTC in very large human brain samples for cytoarchitectonic investigations. The current set-up can process considerable parts of the human brain, such as the occipital lobe, within a 10-day (cytoMASH) or 18-day (combined angio- and cytoMASH) time span. As the sample holders are 3D printed, it would be easy to upscale this set-up even more to accommodate entire sections of human or other large mammalian brains.

### Production of 3D printed optical tissue clearing equipment

Although PP is normally not biodegradable (Arutchelvi et al. 2008), it is a recyclable polymer and its outstanding chemical properties make it ideal for the purpose of custom made tissue clearing hardware (Maddah, H. A. 2016). PP was therefore an obvious choice for the first prototype produced with SLS. This process allows for printing without support structures and therefore gives more freedom with regards to the shape of the print. The prints produced with this method were generally more rigid and the printing resolution was higher, so that the fitting of the components was generally more precise. This higher printing quality is offset, however, by a much higher cost of both the printer itself, and the higher production cost per item (especially when obtained from external companies rather than produced on site). In contradistinction, the quality provided by FDM/FFF printing is sufficient for large-scale prints such as these and comes at much lower cost. FDM printing of prototypes on site greatly reduces the cost by two orders of magnitude as compared to the commercial SLS prints. Given that the printers themselves are cheaper to the same degree, this mode of production seems to be the best choice, especially for smaller laboratories. Once the price for SLS printers is further reduced, this manufacturing process could replace filament printing, given its other advantages. One problem with the FDM/FFF prints is the waste generated by the support structures. Future design improvements could reduce this by, e.g., printing the tower in parts without supports and assembling it later with an organic-solvent resistant adhesive. In general, the great flexibility of 3D printing enables rapid prototyping and adjustments of the size of the containers. Fields such as comparative neuroanatomy, which have to deal with unusual sample sizes (Herculano-Houzel et al. 2014; Marino et al. 2002), might profit from this technology.

### High-throughput pipeline for human brain clearing

One problem when slicing brain tissue is the preservation of its shape, including gyri that are not attached to the main part of the section. Although cutting much thicker slices as compared to traditional histology of several millimetre thickness helps to alleviate this problem, it cannot be fully avoided by this measure alone. To give support to the tissue during cutting, we opted for embedding the samples in 4% agar. Even though the agar becomes very transparent after clearing, it is still visible in the microscopical images (see Fig. 4a) and can produce light scattering. Depending on the LSFM geometry, it can, therefore, be desirable to remove as much agar as possible. Manual removal of the agar is difficult and prone to damaging the cortex. A possible future solution for that could be the use of low-melting point alternatives to agar, such as, low-melting agarose (Iulianella, A. 2017) or alternatively gelatine (Hwang et al. 2006) as an even lower-cost alternative. In these cases, the embedding material could be removed by heating up the sample with warm water. Of course, this would necessitate fixing the tissue in place for imaging before this point. Tissue samples of the size introduced in this chapter necessitate an oblique LSFM geometry (Glaser et al. 2017, 2019; Schueth et al. 2023) because in conventional set-ups the light cannot penetrate to the full lateral extend of the sample. Since the agar is located around the tissue slices, its impact on imaging quality is less severe than for traditional geometries. Whether the embedding material needs to be removed will depend on many factors such as sample thickness, transparency, wavelength of the fluorophore, and tissue type and should therefore be considered on a case-by-case basis.

### Outlook

Although the data presented here are only intended to serve as a proof of concept, it is posited here that OTC and, in some cases, even the labelling, are no longer the limiting factor in the rapidly evolving field of 3D histology. As shown here, hundreds of cm^3^ of brain tissue can be processed simultaneously and in a feasible timeframe. This capacity puts an emphasis on the development of data analysis routines, which can accommodate the large datasets resulting from such samples and process them in a reasonable amount of time. Some image visualization and analysis companies turned their attention to this particular need and several freely available tools have been developed to address some of these issues. Recently solutions have been introduced for multi-view fusion (Amat et al. 2015; Pietzsch et al. 2015), stitching (Bria and Iannello 2012; Hörl et al. 2019), visualization (Pietzsch et al. 2015; Royer et al. 2015), and compression (Amat et al. 2015; Balázs et al. 2017) for large LSFM datasets. Although the imageJ environment (Schindelin et al. 2012) combines some of these tools, there has been no open-source tool available for every processing step until recently. PetaKit5D is one of the first solutions that combines all necessary (pre-)processing steps for oblique LSFM data, including deconvolution, which is suitable for very large datasets (Ruan et al. 2024). We are optimistic that tools such as these, which allow for user-friendly data handling, will improve even more in the near future. These recent and ongoing software developments would significantly lower the threshold for small-scale laboratories to use 3D histology of large post-mortem samples.

In combination with the cost-effective tissue-processing pipeline presented here, which addresses the first steps of any 3D histology workflow, these recent developments open the door towards the routine investigation of the human brain in health and disease at a truly unprecedented scale.

## Supplementary Information

Below is the link to the electronic supplementary material.

Supplementary file1 (MP4 17475 KB)

Supplementary file2 (MP4 13882 KB)

Supplementary file3 (MP4 78709 KB)

Supplementary file4 (STL 201 KB)

Supplementary file5 (STL 679 KB)

Supplementary file6 (STL 510 KB)

Supplementary file7 (STL 1116 KB)

Supplementary file8 (STL 688 KB)

Supplementary file9 (STL 512 KB)

Supplementary file10 (STL 51 KB)

Supplementary file11 (STL 36 KB)

Supplementary file12 (STL 40 KB)

Supplementary file13 (STL 52 KB)

Supplementary file14 (PDF 3216 KB)

## Data Availability

All datasets shown in this publication can be made available upon request to S. Hildebrand (sven.hildebrand@maastrichtuniversity.nl).
