# Supplementary material for: ht-MASH: a high-throughput, cost-effective, and robust protocol for microscopic 3D imaging of human angio- and cytoarchitecture in large human brain samples
Source: Anat Sci Int. 2025 Jul 8;100(4):498–513. doi: 10.1007/s12565-025-00859-w (PMC12513950; doi:10.1007/s12565-025-00859-w)
Supplement: Supplementary file 14 — Supplementary file14 (PDF 3216 KB) [file 12565_2025_859_MOESM14_ESM.pdf]

Supplementary figures and files ht-MASH: A High-Throughput, Cost-Effective, and Robust Protocol for 3D Imaging of Human Angio- and Cytoarchitecture in Large Human Brain Samples

Supplementary Video 1: Resliced volume of occipital lobe 1, slice 6. The color has been inverted to closer mimic a conventional Nissl staining and the contrast and brightness has been adjusted automatically for each image plane. Otherwise, no further image processing has been performed.

Supplementary Video 2: Resliced volume of occipital lobe 2, slice 6. The color has been inverted to closer mimic a conventional Nissl staining and the contrast and brightness has been adjusted automatically for each image plane. Otherwise, no further image processing has been performed.

Supplementary Video 3: 3D volume rendering of an occipital lobe 3 sample. Cell bodies are pseudo-coloured in green and blood vessels in magenta.

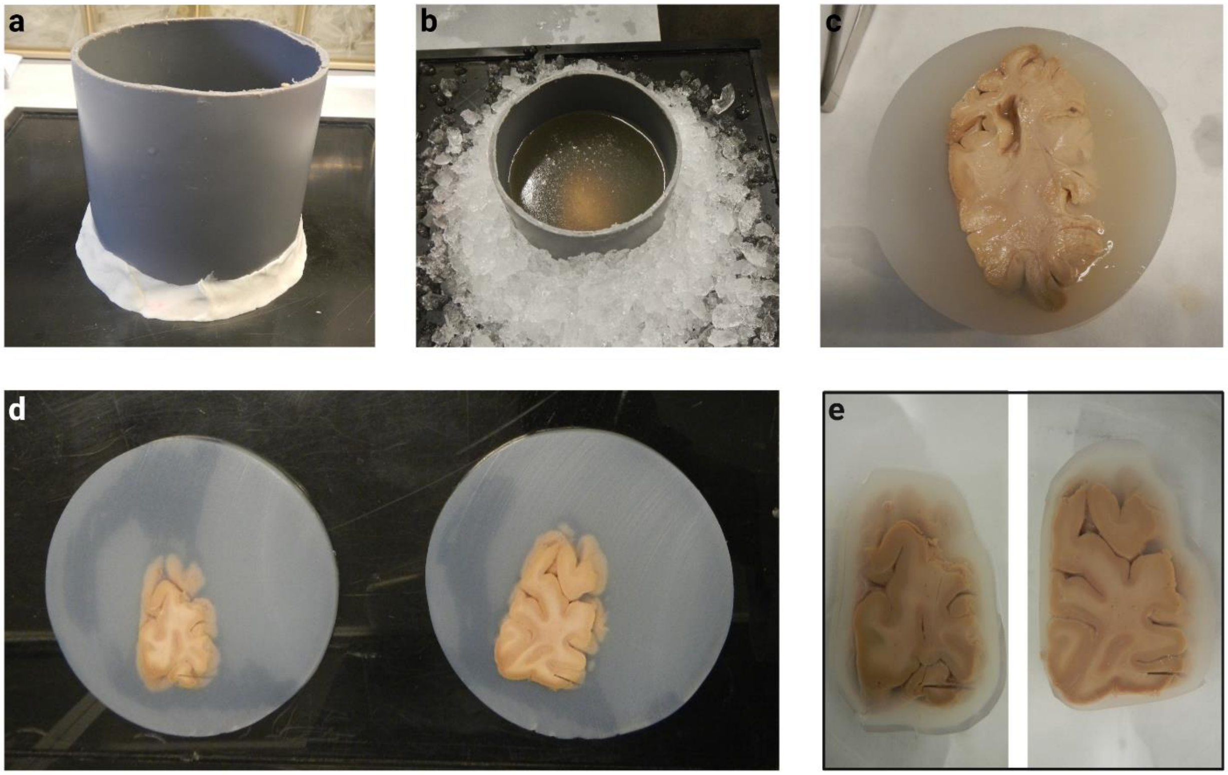

Supplementary Figure 1: Embedding of human occipital lobe in 4% agar. (**a**) A 12 cm plastic pipe cut to size is fixed on a plastic plate with putty. (**b**) The tissue is placed inside and the pipe filled with hand warm 4% agar solution. Crushed ice is added for faster solidification of the agar. (**c**) Embedded occipital lobe 3. (**d** and **e**) two 3 mm slices after sectioning with the rotary meat cutter before (d) and after (e) trimming the agar.

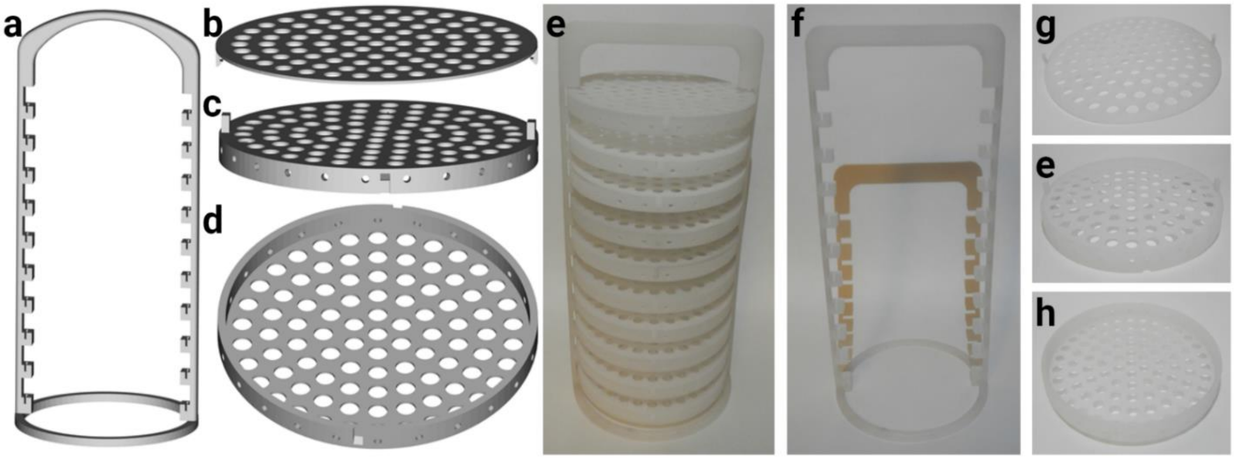

Supplementary Figure 2: Clearing tower and sample holder prototypes for SLS printing. (**a**) The tower can fit up to 10 sample holder discs at once. In order to provide support to the sample holders (**b** – **d**), grooves have been introduced into which the feet of the sample holders can be placed (see topside in **c**). (**e** -**h**) Final prototype of commercial SLS printing.

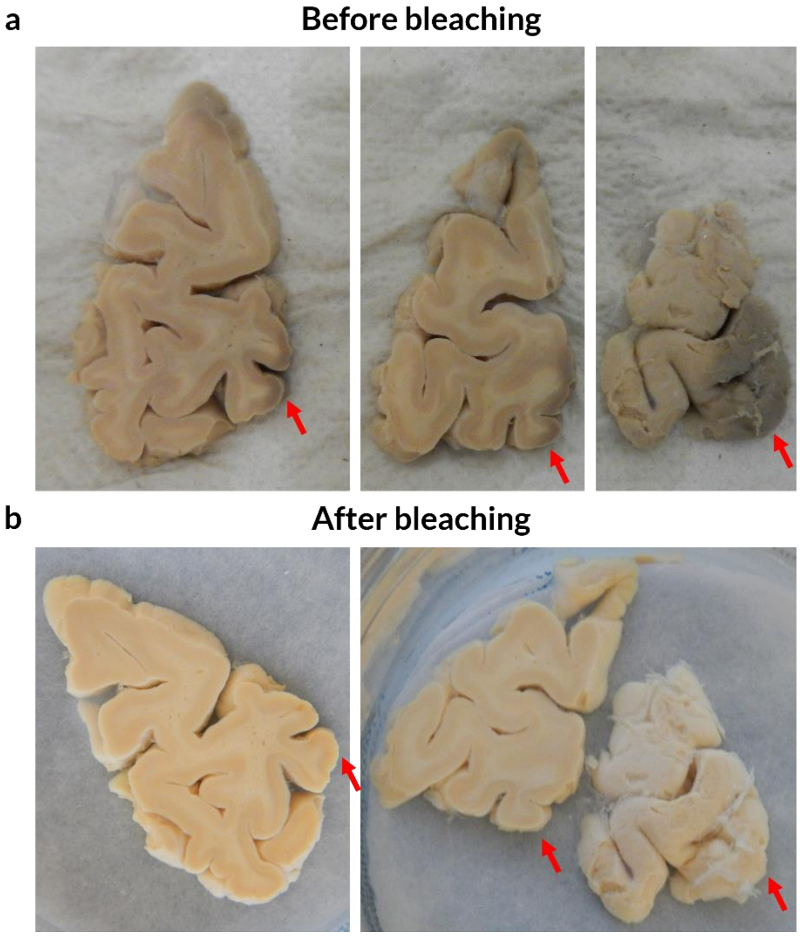

Supplementary Figure 3: Macroscopic evaluation of double bleaching efficacy. (**a**) Three consecutive samples of occipital lobe 4 before OTC processing started. Note the darker areas, which are most likely postmortem accumulations of blood in the occipital pole (red arrows). (**b**) The same samples after the double bleaching procedure (first 5% H_2O_2 in methanol, second 50% aqueous potassium disulfite).

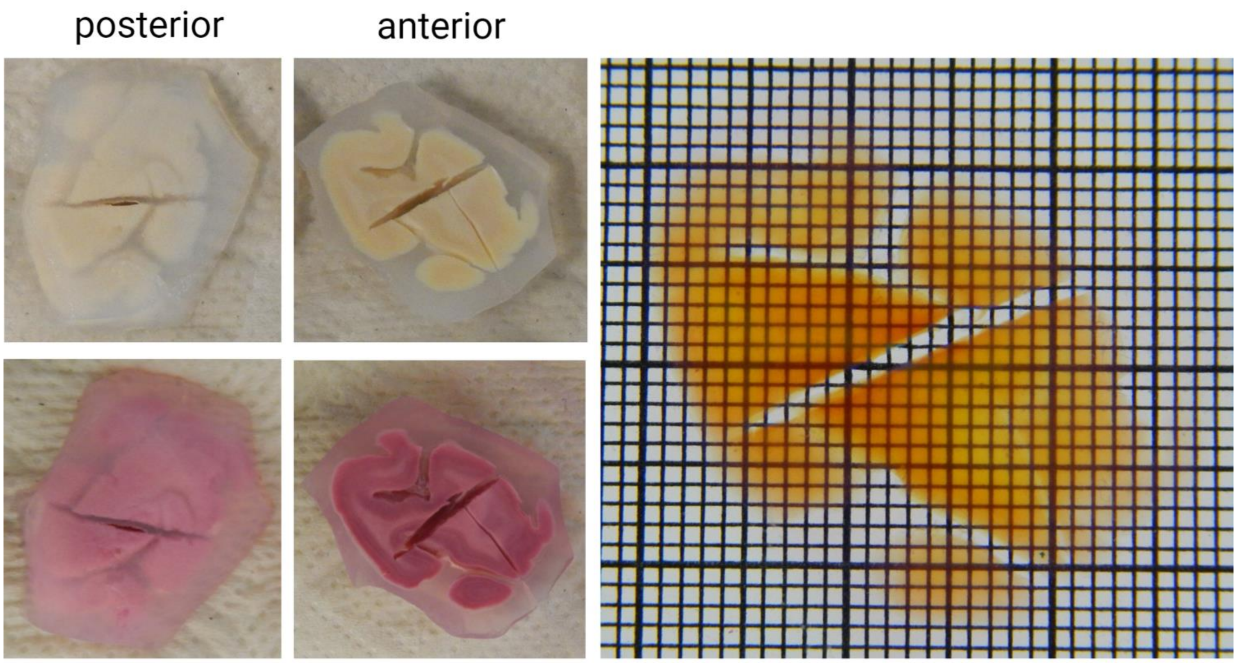

Supplementary Figure 4: Occipital lobe 1, slice 1 (posterior to anterior). Posterior views of the sample after bleaching (top) and staining (bottom) are shown in the left, anterior views on in the middle. The cleared and RI-matched sample is shown on the right (Grid: 1x1 mm smallest squares; 10x10 mm bold squares).

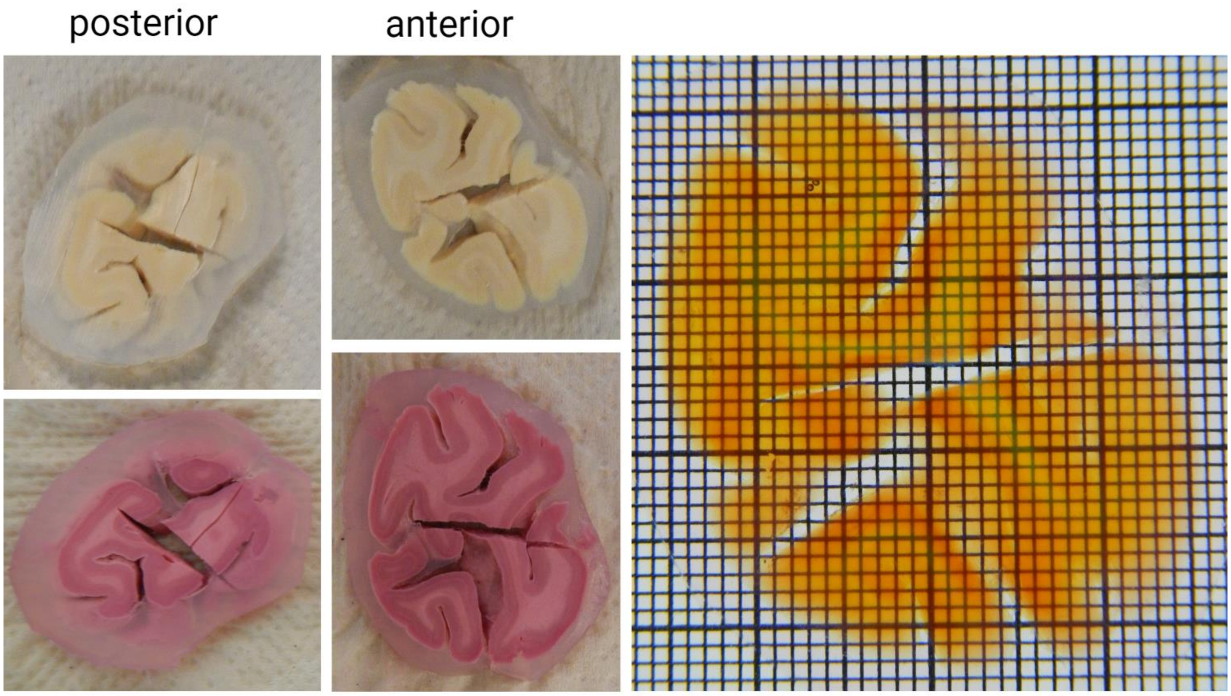

Supplementary Figure 5: Occipital lobe 1, slice 2 (posterior to anterior). Posterior views of the sample after bleaching (top) and staining (bottom) are shown in the left, anterior views on in the middle. The cleared and RI-matched sample is shown on the right (Grid: 1x1 mm smallest squares; 10x10 mm bold squares).

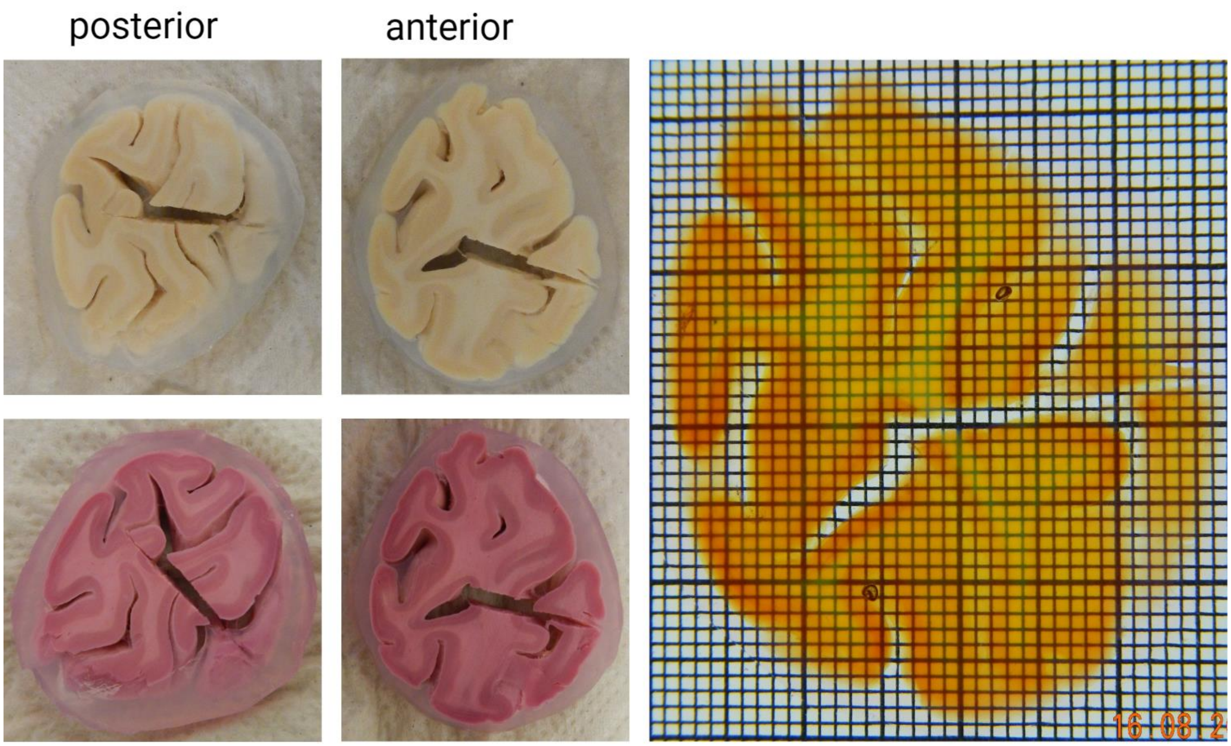

Supplementary Figure 6: Occipital lobe 1, slice 3 (posterior to anterior). Posterior views of the sample after bleaching (top) and staining (bottom) are shown in the left, anterior views on in the middle. The cleared and RI-matched sample is shown on the right (Grid: 1x1 mm smallest squares; 10x10 mm bold squares).

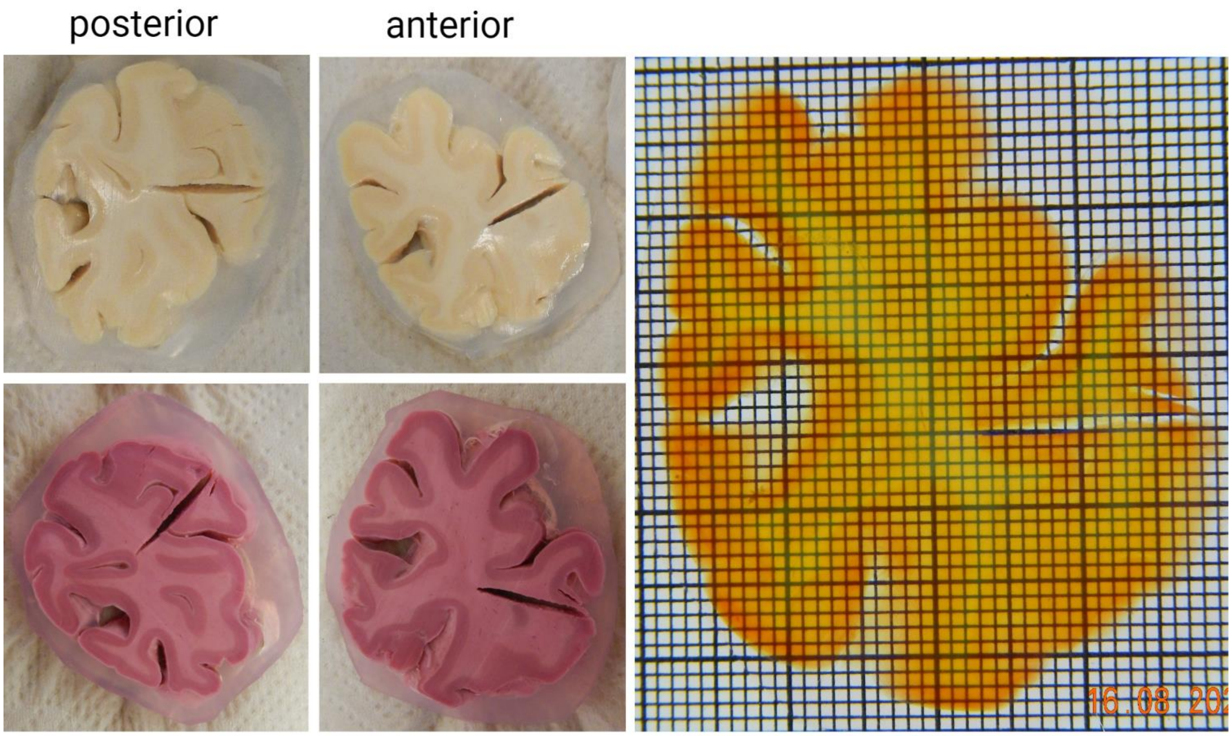

Supplementary Figure 7: Occipital lobe 1, slice 4 (posterior to anterior). Posterior views of the sample after bleaching (top) and staining (bottom) are shown in the left, anterior views on in the middle. The cleared and RI-matched sample is shown on the right (Grid: 1x1 mm smallest squares; 10x10 mm bold squares).

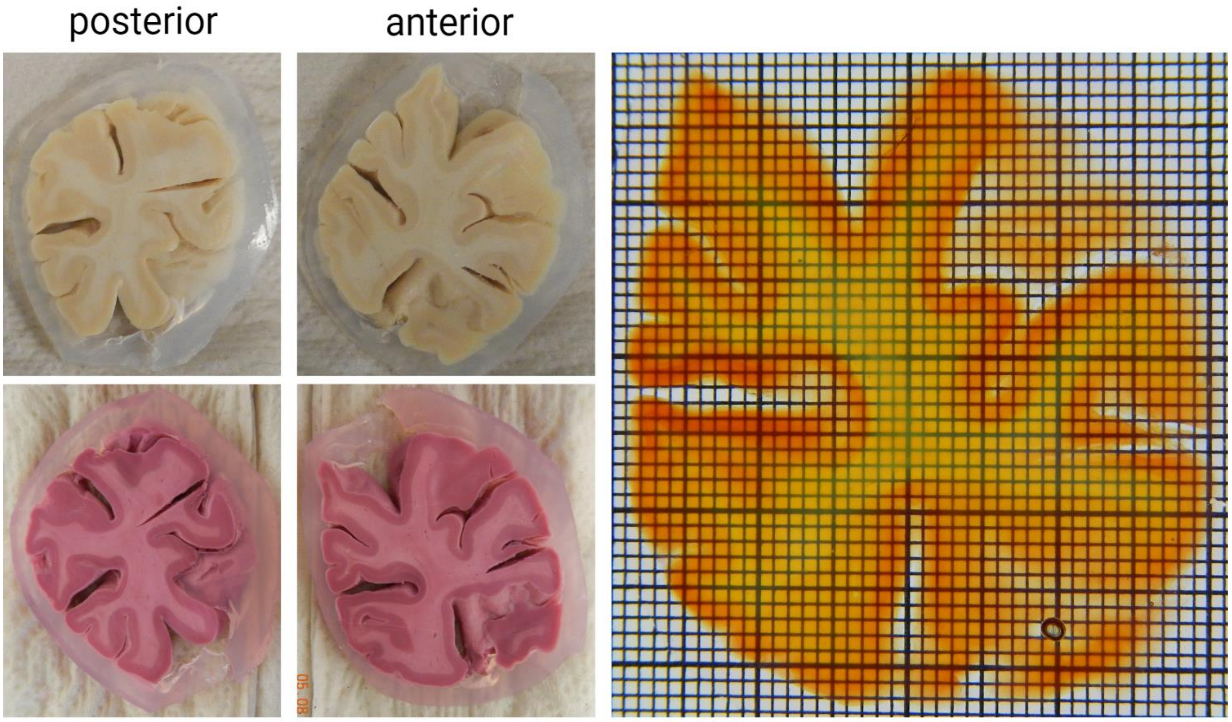

Supplementary Figure 8: Occipital lobe 1, slice 5 (posterior to anterior). Posterior views of the sample after bleaching (top) and staining (bottom) are shown in the left, anterior views on in the middle. The cleared and RI-matched sample is shown on the right (Grid: 1x1 mm smallest squares; 10x10 mm bold squares).

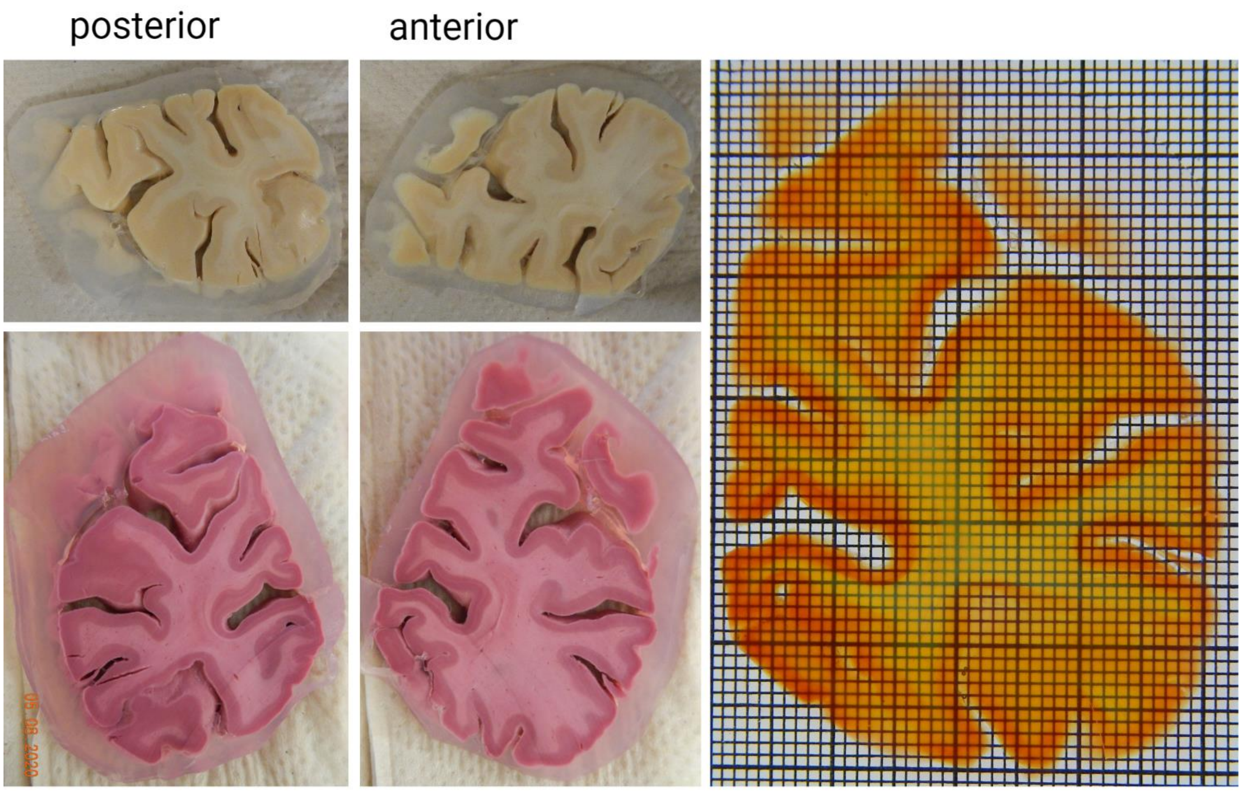

Supplementary Figure 9: Occipital lobe 1, slice 6 (posterior to anterior). Posterior views of the sample after bleaching (top) and staining (bottom) are shown in the left, anterior views on in the middle. The cleared and RI-matched sample is shown on the right (Grid: 1x1 mm smallest squares; 10x10 mm bold squares).

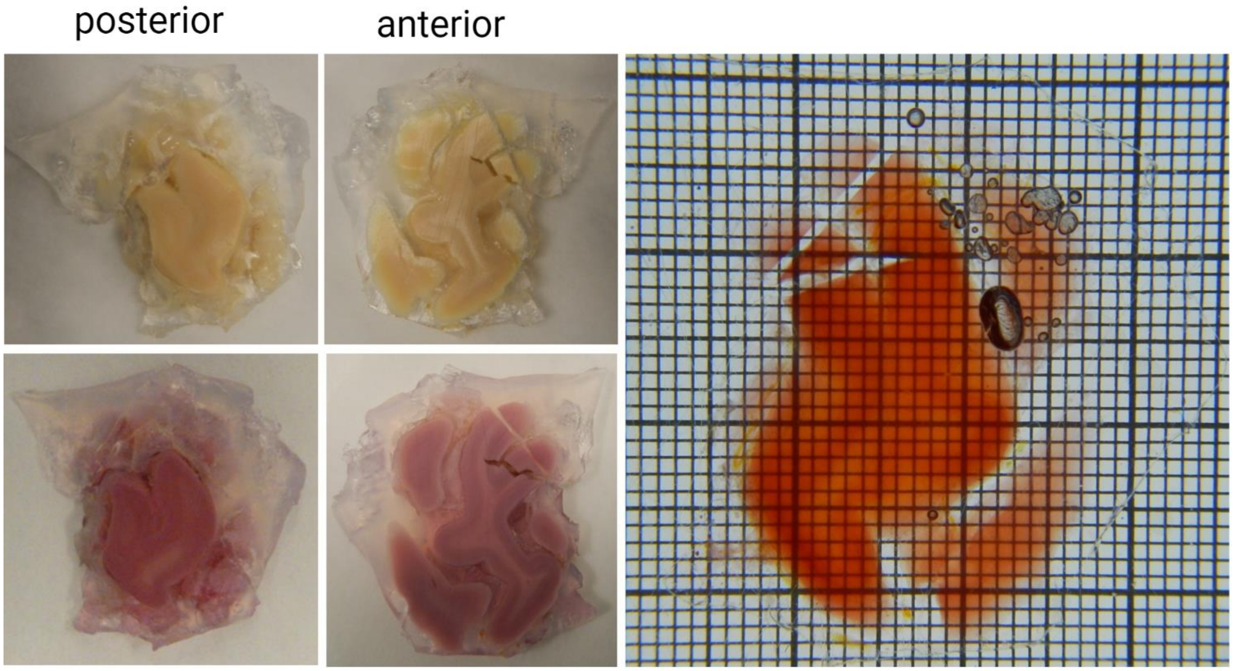

Supplementary Figure 10: Occipital lobe 2, slice 1 (posterior to anterior). Posterior views of the sample after bleaching (top) and staining (bottom) are shown in the left, anterior views on in the middle. The cleared and RI-matched sample is shown on the right (Grid: 1x1 mm smallest squares; 10x10 mm bold squares).

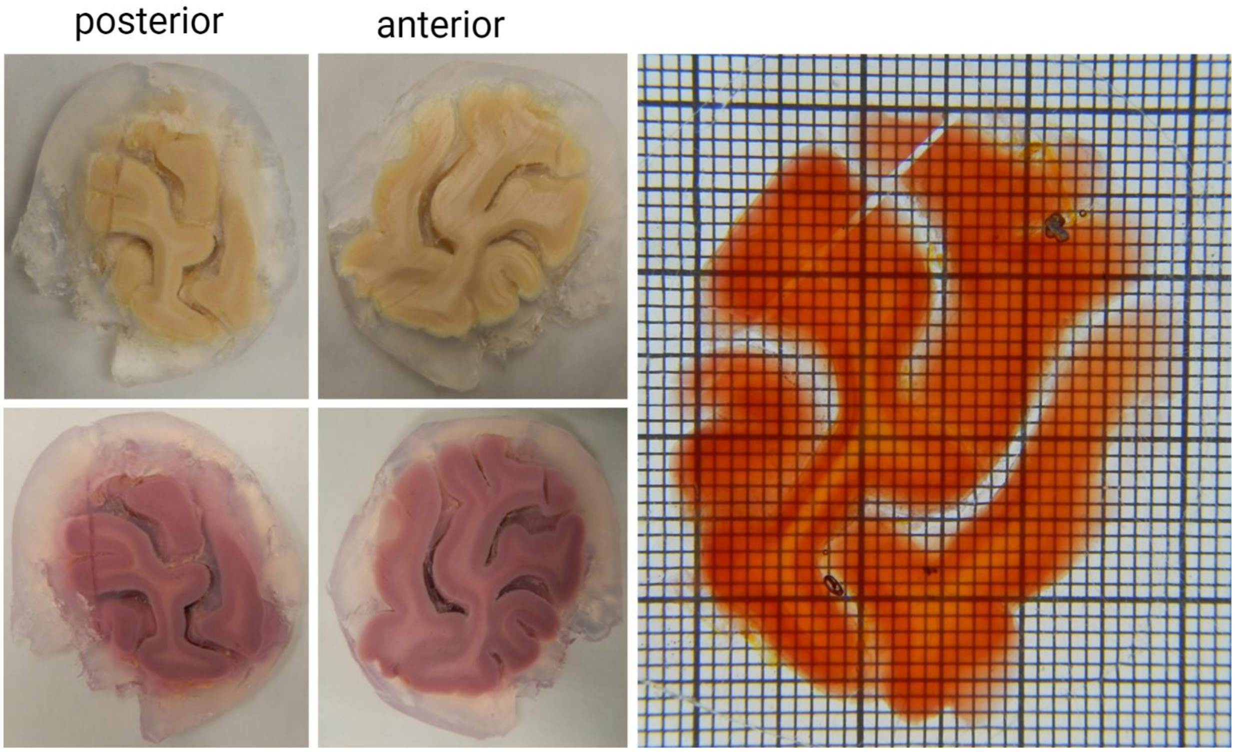

Supplementary Figure 11: Occipital lobe 2, slice 2 (posterior to anterior). Posterior views of the sample after bleaching (top) and staining (bottom) are shown in the left, anterior views on in the middle. The cleared and RI-matched sample is shown on the right (Grid: 1x1 mm smallest squares; 10x10 mm bold squares).

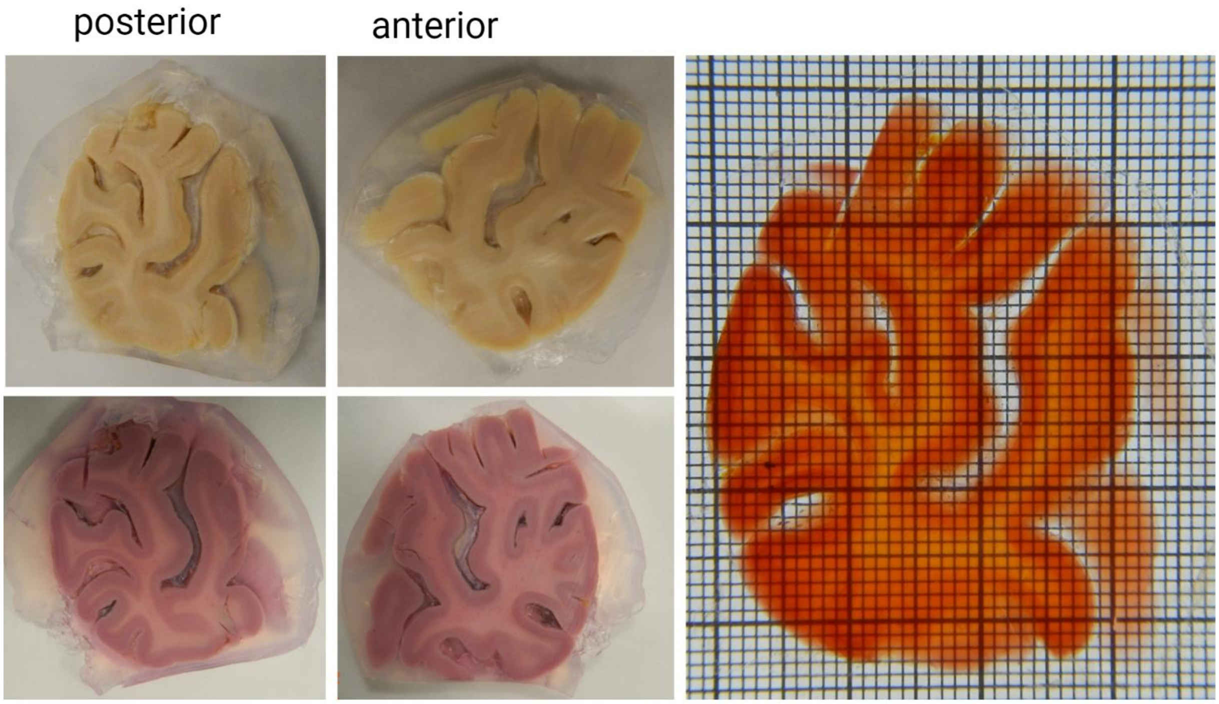

Supplementary Figure 12: Occipital lobe 2, slice 3 (posterior to anterior). Posterior views of the sample after bleaching (top) and staining (bottom) are shown in the left, anterior views on in the middle. The cleared and RI-matched sample is shown on the right (Grid: 1x1 mm smallest squares; 10x10 mm bold squares).

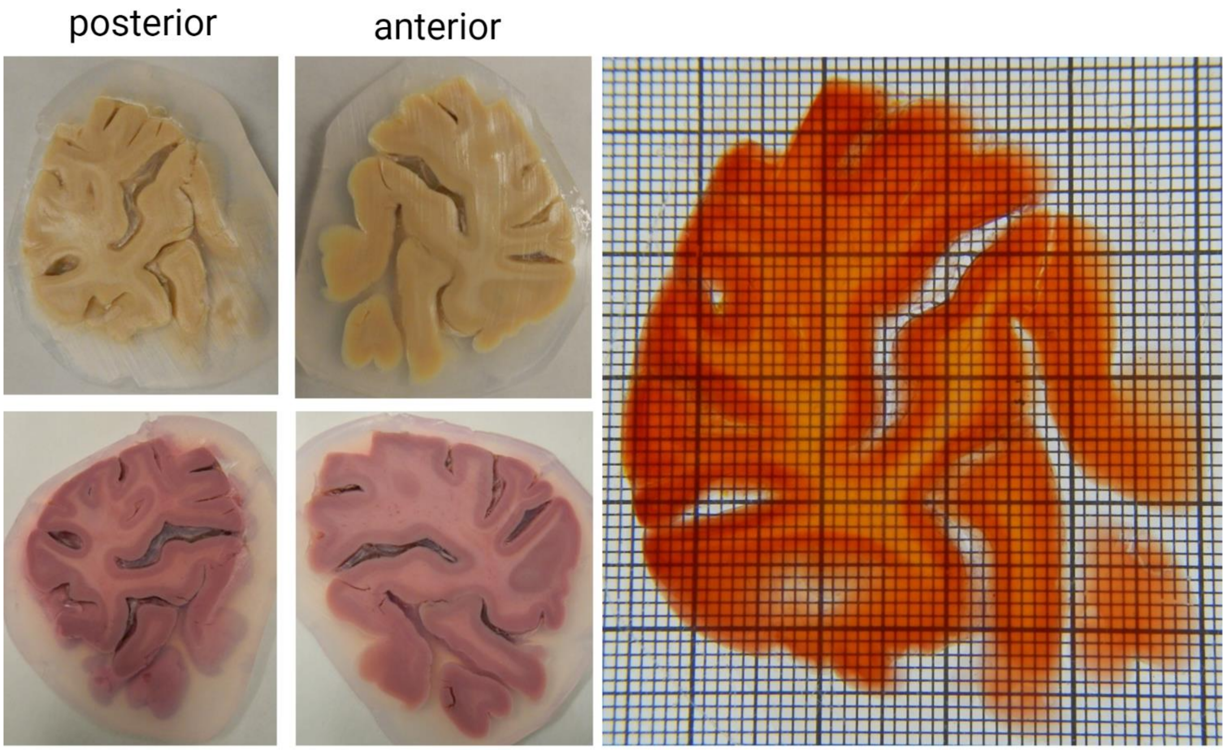

Supplementary Figure 13: Occipital lobe 2, slice 4 (posterior to anterior). Posterior views of the sample after bleaching (top) and staining (bottom) are shown in the left, anterior views on in the middle. The cleared and RI-matched sample is shown on the right (Grid: 1x1 mm smallest squares; 10x10 mm bold squares).

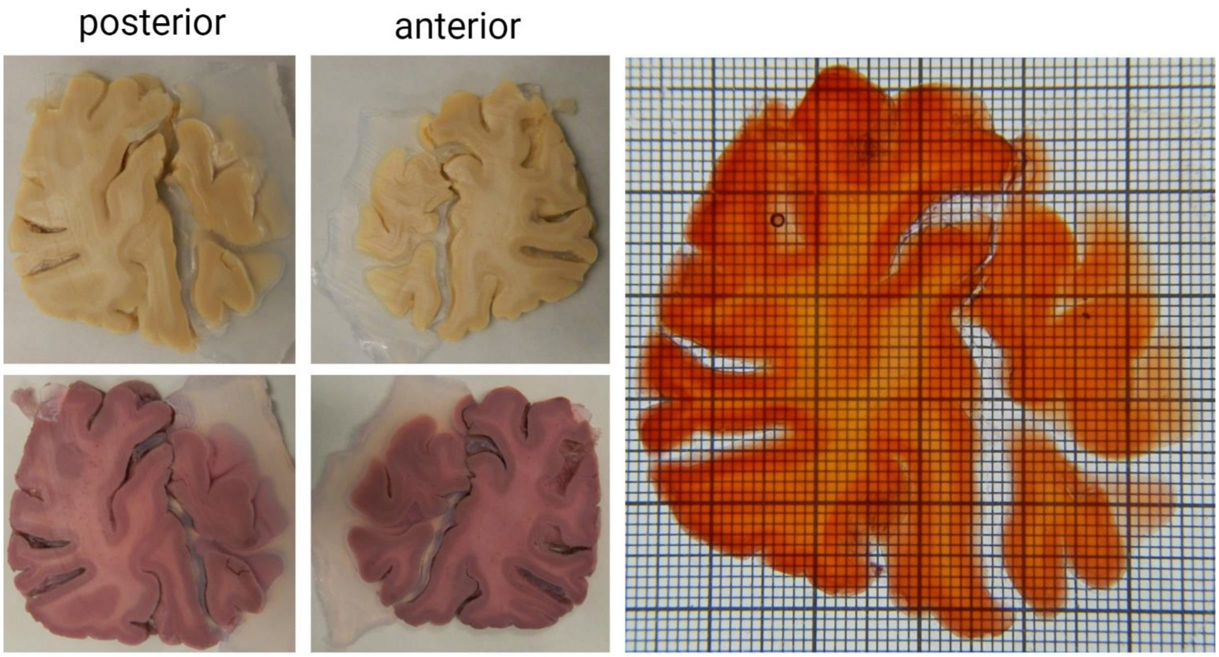

Supplementary Figure 14: Occipital lobe 2, slice 5 (posterior to anterior). Posterior views of the sample after bleaching (top) and staining (bottom) are shown in the left, anterior views on in the middle. The cleared and RI-matched sample is shown on the right (Grid: 1x1 mm smallest squares; 10x10 mm bold squares).

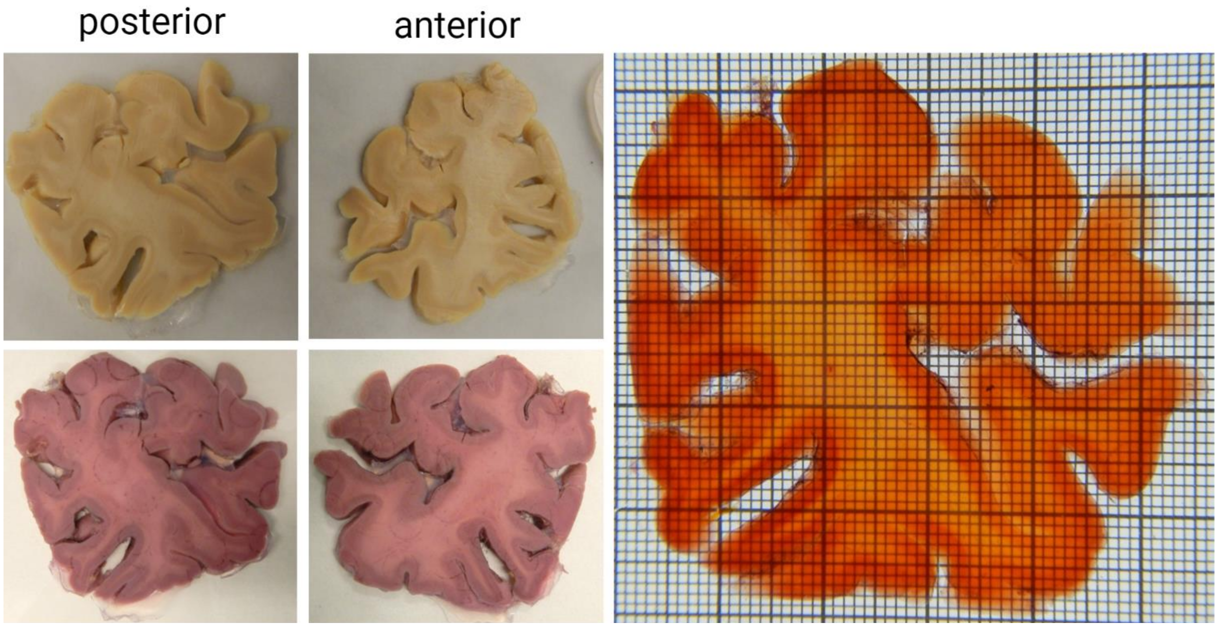

Supplementary Figure 15: Occipital lobe 2, slice 6 (posterior to anterior). Posterior views of the sample after bleaching (top) and staining (bottom) are shown in the left, anterior views on in the middle. The cleared and RI-matched sample is shown on the right (Grid: 1x1 mm smallest squares; 10x10 mm bold squares).

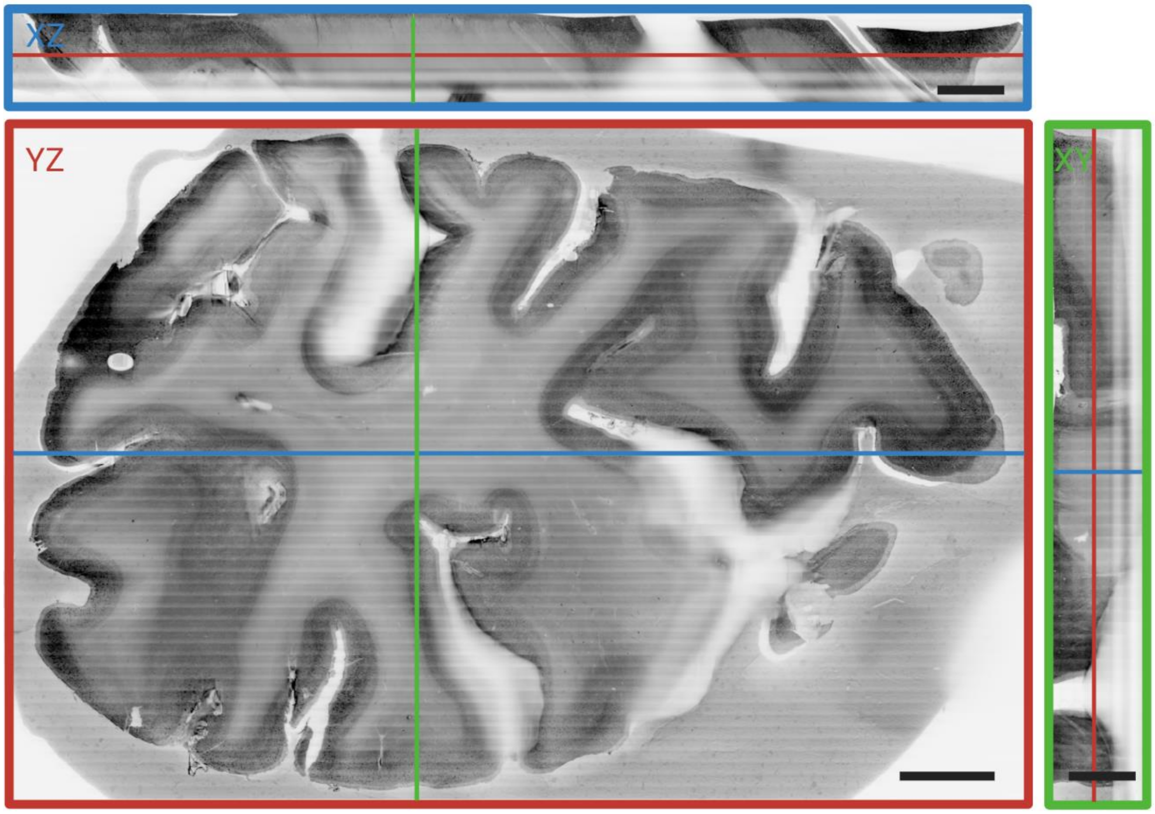

Supplementary Figure 16: Orthogonal views of the anterior most slice of occipital lobe 1. Single plane views across each axis to demonstrate the label penetration and quality. Scale bars: YZ: 5 mm; XY and XZ: 3.5 mm.

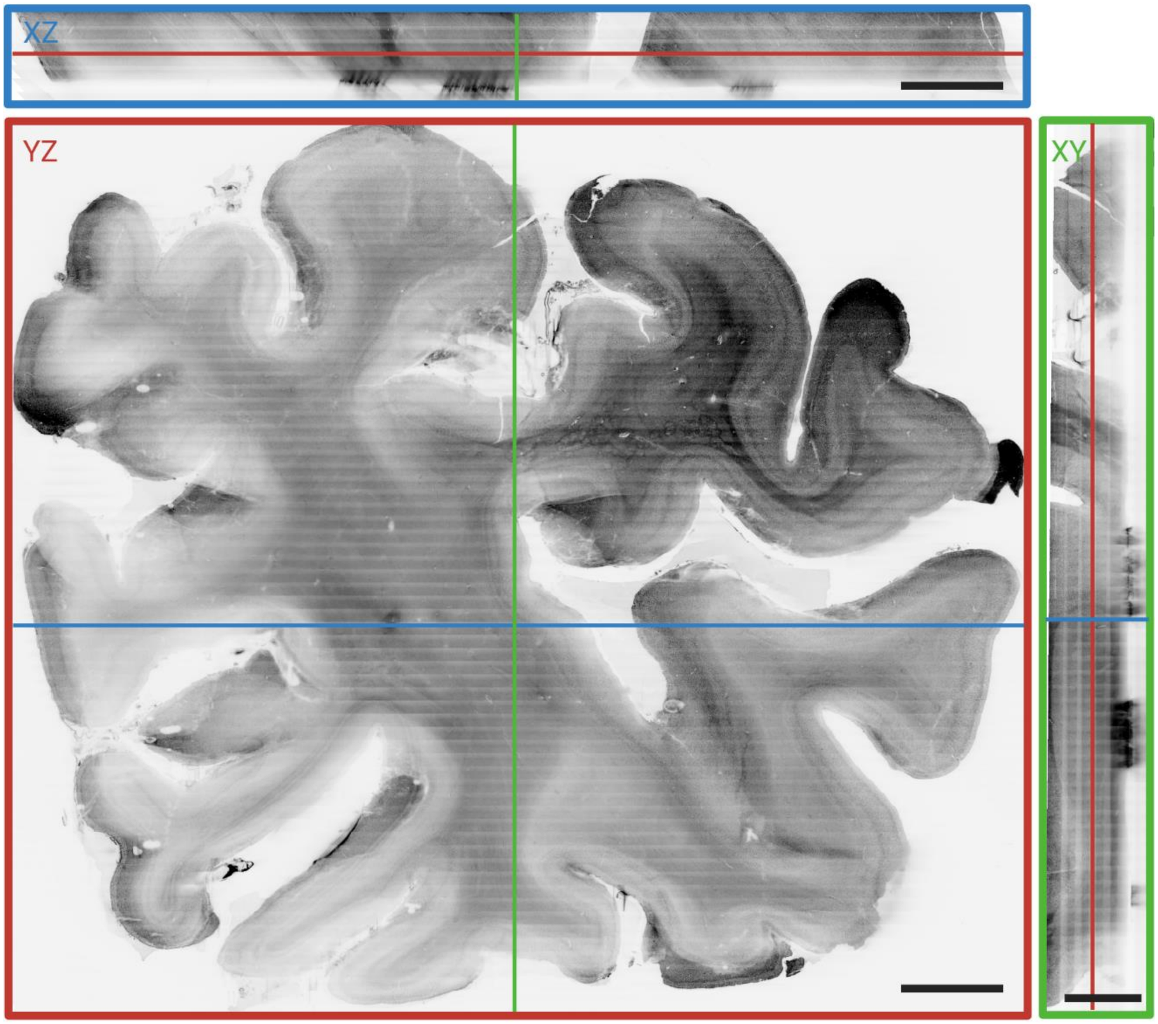

Supplementary Figure 17: Orthogonal views of the anterior most slice of occipital lobe 2. Single plane views across each axis to demonstrate the label penetration and quality. Scale bars: YZ and XZ: 5 mm; XY: 3.5 mm.

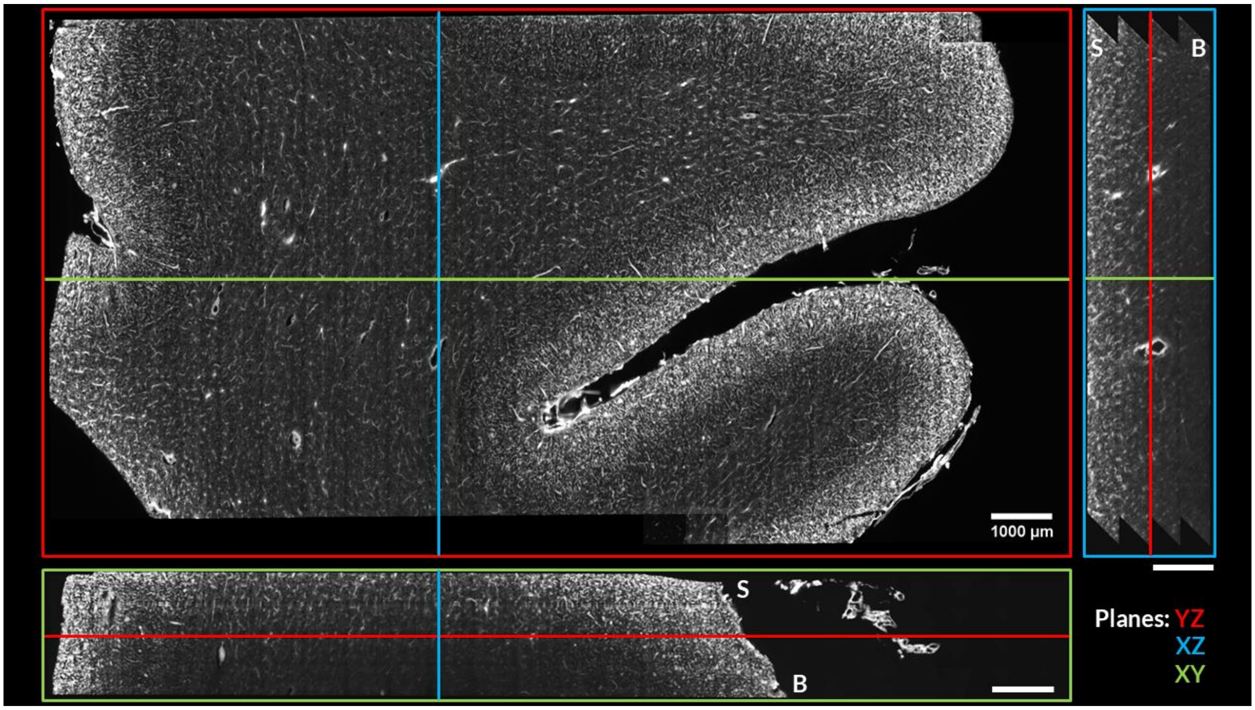

Supplementary Figure 18: Orthogonal views of the anterior most slice of occipital lobe 3. Single plane views across each axis to demonstrate the label penetration and quality. Letters “S” denote the tissue surface and letters “B” the tissue bottom. There is a visible overall falloff in intensity. However, this is not related to the label penetration (see homogenous vessel labelling in the center YZ slice (red), but rather to light scattering with increasing tissue depth. Scale bars: 1 mm.
